# Ablation of Iron Regulatory Protein 2 produces a neurological disorder characterized by motor, somatosensory, and executive dysfunction in mice

**DOI:** 10.1016/j.crneur.2024.100136

**Published:** 2024-08-10

**Authors:** Christina Porras, Hayden Olliviere, Sean P. Bradley, Alice M. Graham, Yogita Chudasama, Tracey A. Rouault

**Affiliations:** a National Institute of Child Health and Development, Section on Human Iron Metabolism, USA; b National Institute of Mental Health, Rodent Behavioral Core, USA; c National Institute of Mental Health, Section on Behavioral Neuroscience, Bethesda, MD 20892, USA

**Keywords:** Iron metabolism, Neurodegeneration, Perseveration, Cognitive flexibility, Iron deficiency

## Abstract

Iron is an important cofactor for many proteins and is used to create Fe-S clusters and heme prosthetic groups that enzymes use to catalyze enzymatic reactions. Proteins involved in the import, export, and sequestration of iron are regulated by Iron Regulatory Proteins (IRPs). Recently, a patient with bi-allelic loss of function mutations in IREB2 leading to the absence of IRP2 protein was discovered. The patient failed to achieve developmental milestones and was diagnosed with dystonic cerebral palsy, epilepsy, microcytic hypochromic anemia, and frontal lobe atrophy. Several more IREB2 deficient patients subsequently identified manifested similar neurological problems. To better understand the manifestations of this novel neurological disease, we subjected an Irp2-null mouse model to extensive behavioral testing. Irp2-null mice had a significant motor deficit demonstrated by reduced performance on rotarod and hanging wire tests. Somatosensory function was also compromised in hot and cold plate assays. Their spatial search strategy was impaired in the Barnes maze and they exhibited a difficulty in flexibly adapting their response in the operant touchscreen reversal learning task. The latter is a cognitive behavior known to require an intact prefrontal cortex. These results suggest that loss of Irp2 in mice causes motor and behavioral deficits that faithfully reflect the IREB2 patient's neurodegenerative disorder.

## Highlights

• Iron Regulatory Protein 2 (Irp2) regulates iron cellular homeostasis.
• Patients with mutations of the Irp2 gene (IREB2) present with dystonia, anemia, epilepsy, and frontal lobe atrophy.
• Irp2 null mice show functional iron deficiency in neurons similar to Irp2 patients.
• Irp2 null mice display motor, somatosensory and frontal cortex deficits.

## Graphical abstract

**Figure:**
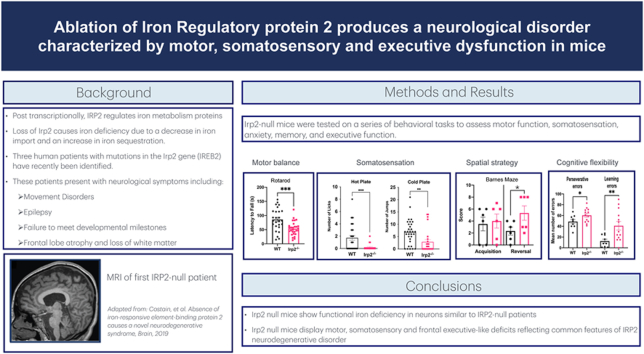

## 1 Introduction

Maintenance of cellular iron homeostasis is a critical process which when disrupted interferes with iron's role in various processes including mitochondrial respiration, DNA synthesis, hemoglobin synthesis, the formation of iron sulfur clusters, myelin maturation and neurotransmitter synthesis (Ghosh et al., 2015; Porras and Rouault, 2022). Iron homeostasis disruption is implicated in several neurodegenerative conditions and recent work on iron metabolism regulation has focused on understanding this connection (Rouault, 2013; Maio et al., 2021, 2022).

Iron Response Proteins 1 and 2 are necessary RNA-binding proteins for regulating proteins involved in iron import, export, and sequestration. IRP1 and IRP2 are ubiquitously expressed, but IRP2 expression in the CNS is relatively high compared to IRP1 (Meyron-Holtz et al., 2004). During high iron conditions an E3 ubiquitin ligase complex containing the F-box protein, FBXL5 initiates the rapid degradation of IRP2. During low iron or hypoxic conditions, FBXL5 loses its ability to bind to IRP2 because of loss of the FeS cluster from iron deficiency or failure of the FeS to undergo oxidation (Rouault and Maio, 2020). IRP2 is thus stabilized and binds to RNA stem-loop structures called Iron Response Elements (IREs) on the mRNA transcripts of iron metabolism proteins (Pantopoulos, 2004). Iron metabolism proteins involved in iron storage (ferritin) and export (ferroportin 1, FPN1) contain an IRE on the 5′UTR of their mRNA transcripts; thus, binding of IRP2 prevents the initiation of translation and downregulates expression of the proteins encoded by these transcripts. Proteins involved in iron import (transferrin receptor 1, TfR1, and divalent metal transporter 1, DMT1) have one of more IREs in the 3′UTR of their mRNA transcripts and are then protected from degradation by IRP2 binding. Therefore, in response to low iron conditions, IRP2 coordinates the expression of iron metabolism proteins to increase intracellular iron conditions and maintain iron homeostasis (Hentze et al., 1987; Casey et al., 1988; Dandekar et al., 1991; Kim et al., 1996; Abboud and Haile, 2000; Donovan et al., 2000; Garrick et al., 2003; Sanchez et al., 2007).

Recently the first IRP2-null human patient was discovered by clinical exome sequencing which identified bi-allelic loss-of-function mutations in IREB2 resulting in the absence of IRP2. The patient was diagnosed with dystonic cerebral palsy and developed a movement disorder. Brain MRIs revealed progressive cerebral atrophy and white matter loss particularly in the frontal regions. The patient failed to meet developmental milestones such as learning to walk or speak. Patient lymphoblasts had a reduction in TfR1 expression and an increase in ferritin expression consistent with iron metabolism misregulation (Costain et al., 2019). Two other IRP2-null patients have since been identified with very similar symptoms to the original patient (Cooper et al., 2019; Maio et al., 2022; Porras and Rouault, 2022).

The discovery of three IRP2-null patients has resolved a controversy that arose around the neurodegenerative phenotype detailed in the original Irp2^−/−^ mouse model created by the Rouault Lab (LaVaute et al., 2001; Jeong et al., 2011). This mouse model developed a progressive neurodegenerative disorder observable by six months of age that was characterized by abnormal gait, ataxia, kyphosis, tremor, postural abnormalities, and poor motor coordination. Ferric iron stains indicated that the mice were accumulating ferritin-bound iron, and amino cupric silver stains showed axon degeneration in white matter tracks correlating with the iron accumulation. The accumulation of ferritin-bound iron was attributed to an increase in the expression ferritin and a decrease in the iron importer protein TfR1 causing a functional iron deficiency. Mitochondrial function was also impaired in the Irp2^−/−^ mice (LaVaute et al., 2001; Jeong et al., 2011). Other models created by the Hentze group (Galy et al., 2004, 2006) and the Leibold group (Zumbrennen-Bullough et al., 2014) did not reliably exhibit the behavioral and biochemical phenotype typically observed in the patient population. Additionally, the breadth of symptoms observed in the IRP2-null patients has raised the possibility that deficits in the cognitive/behavioral domain may have been missed in the initial phenotypic characterization of the Irp2^−/−^ mouse model. Due to the mildness of the phenotype compared with the human patients, the Irp2^−/−^ mouse model may also offer the opportunity to discover additional symptoms that could be present in human patients that retain some IRP2 functionality as well as contribute to the understanding of the consequences of iron deficiency more generally. The following study has attempted to explore these possibilities and establish a comprehensive motor, sensory and behavioral characterization of mice lacking iron regulatory protein 2.

## 2 Materials and methods

### 2.1 Mice

All experimental procedures were approved by NICHD Institutional Animal Care and Use Committee, in accordance with the NIH guidelines for the use of animals. Irp2 ablation was accomplished as described previously (LaVaute et al., 2001). These mice were then backcrossed to C57Bl/6J mice to eliminate the mixed genetic background of the original model. Unless otherwise noted, all mice had ad libitum access to food and water. All testing was completed during the light cycle of the mice. Mice were allowed to habituate to the testing rooms for a minimum of 10 min, and in between mice each platform/apparatus was cleaned with 70% ethanol.

### 2.2 Motor control and balance: rotarod

In total 54 genotype, sex, and age-matched mice were tested: WT (n = 26), Irp2^−/−^ (n = 28), male (n = 20), female (n = 34), young (4–6 months old, n = 24), middle aged (10–12 months old, n = 30). Mice were placed on a rotarod (IITC Life Science) set to increase from 4 rpm to 40 rpm over 5 min. Each mouse completed two sets of three runs over two days for a total of six trials. The number of trials was determined based on the number of trials it took for wildtype mice to learn they can grip the rotarod which artificially increased the latency to fall. Latency to fall was recorded and averaged over the six trials. One mouse jumped from the rotarod which led us to exclude this single trial, leaving a total of five trials for this animal.

An unpaired *t*-test (GraphPad Prism) was used for initial analysis of rotarod performance (a Welch's correction for comparison of genotypes). This was followed by a three-way ANOVA (GraphPad Prism) to examine the effects of genotype, sex, and age. Multiple comparisons of means that differ by only one factor was done with a Šídák correction. To control for weight, an ANCOVA model (SPSS) was used with weight as the covariate; genotype, age, and sex as fixed factors; and latency to fall as the dependent variable. A Bonferroni correction was used for multiple comparisons.

### 2.3 Grip strength: hang test

The hang test was used to measure grip strength and performed as described previously (LaVaute et al., 2001). Age (4–6 months old: n = 24; 11–12 months old: n = 33) and sex-matched WT (n = 27) and Irp2^−/−^ (n = 30) mice were placed on a wire grid which was shaken lightly to encourage the mice to grip the bars. The grid was then flipped upside down for 60 s and the latency to fall was recorded. Basic grip strength was analyzed using unpaired *t*-test (GraphPad Prism). To control for weight, an ANCOVA model (SPSS) was used with weight as the covariate; genotype and sex as fixed factors; and latency to fall as the dependent variable. A Bonferroni correction was used for multiple comparisons.

### 2.4 Somatosensory perception: hot and cold plate assay

Age (3–10 months old; avg 7 months old) and sex-matched WT (n = 32) and Irp2^−/−^ (n = 26) mice were placed on a hot (52 °C) and cold (0 °C) plate (Ugo Basile) for 30 s. Each response was recorded and analyzed (ANY-maze) by a blind observer who detected the occurrence of hind-paw licks (hot) and jumps (cold). Latency for the first occurrence of both events was also determined with a latency of 30 s used in the absence of the event during the assay. Data were analyzed using unpaired *t*-test (GraphPad Prism). A Welch's correction was used for comparison of latency to lick and lick number on the hot plate due to a significant difference in variance between the two groups (p < 0.0001).

### 2.5 Spontaneous locomotion: open field test

Age (4–6 months old: n = 24; 11–12 months old: n = 11) and sex (male: n = 12; female: n = 23)-matched mice (WT n = 16; Irp2^−/−^ n = 19) were placed in a rectangular, clear plexiglass box (40x25 × 20 cm) for 60 min in the dark once daily for three days. The Photobeam Activity System (PAS)-Open Field system (San Diego Instruments) software was used to track mouse locomotion. Beam brakes were averaged over all three trials. Any trials where a mouse escaped the apparatus were excluded.

The PAS software distinguishes between ambulatory and fine movement in the center and periphery of the apparatus. An unpaired *t*-test was used to compare total beam brakes for each measurement. Periphery ambulatory movement beam brakes were binned into 10-min intervals for a two-way RM ANOVA analysis with a Šídák correction for multiple comparisons (GraphPad Prism).

### 2.6 Thigmotaxis/anxiety: open field test

A second open field test was performed using a larger, square open field (44x44 × 39 cm). Age (4–6 months old) and sex matched mice (n = 8) were placed in the open field for 30 min in dim lighting. Video footage was analyzed (ANY-maze) and performance metrics were averaged over all three trials. The “center” zone was created digitally and included a quarter of the open field surface area (22 × 22 cm) creating a periphery zone 11-cm-wide around the center (Fig. S3). An unpaired *t*-test was used to compare WT and Irp2^−/−^ performance.

### 2.7 Anxiety: elevated zero maze

The same mice used in the previous test (2.6) were also subjected to an elevated-zero maze test consisting of two open and closed sections. Mice were placed in an open section of the maze alternatingly facing one of four closed section entrances. Mice were allowed to freely explore the maze for 5 min with the lights on. Video footage of the test was analyzed (ANY-maze) to assess the percentage of time spent in the open sections of the maze. Unpaired t-tests were used to compare WT and Irp2^−/−^ mice performance.

### 2.8 Learning and memory: Y-maze

Age (4–6 months old) and sex-matched WT (n = 11) and Irp2^−/−^ (n = 13) mice were placed in the center of an opaque, open Y-maze consisting of three identical arms measuring 40x13 × 10 cm spaced 120° from each other. Trials lasted for 10 min and were completed on three consecutive days. Performance was captured and analyzed (ANY-maze) by a blind observer noting entry into each arm (entry defined as passage of all four paws of the mice moving past the entry of each arm). The ANY-maze motion tracking software was used to track average distance travelled, speed, number of line crossings, and time spent immobile on average over three trials. Each set of three entries where the mouse visited each arm was counted as a triad. An overlapping technique was used such that a pattern of A-C-B-C-A consisted of two triads. The number of spontaneous alternations was expressed as a percentage according to the following formula:

$$\text{Alternation}\;\text{index}=\frac{⋕\text{of}\;\text{alternations}}{⋕\text{of}\;\text{arm}\;\text{Entries}-2}\times 100\%$$

### 2.9 Learning and memory: Barnes Maze

The Barnes Maze experimental protocol was adapted from Gawel et al. (2019) and Pitts (2018). An illustration of the Barnes Maze layout is included in Fig. S5. Age (5–7 months old) and sex-matched WT and Irp2^−/−^ mice (n = 6) completed the following stages.

#### 2.9.1 Habituation (Day 0)

Each mouse was placed in the center of the maze under a container for 30 s to randomize initial heading of the mouse. The cup was then removed and the experimenter gently guided the mouse to the escape box. The mouse remained inside the escape box for 30 s, and then was returned to their holding cage. After a 2-min inter-trial interval, the trial was repeated except that the mouse was allowed to explore the maze for 3 min before the experimenter gently guided the mouse to the escape box.

#### 2.9.2 Acquisition training (Day 1–5)

Each mouse was placed in the center of the maze under a container for 30 s. The container was then removed, the experimenter left the testing room, and the mouse was allowed to freely explore the maze for 3 min. If the mouse entered the escape box, the box was covered, and the mouse remained inside for 30 s. Otherwise after 3 min the mouse was gently guided to the escape box by the experimenter, and the mouse remained inside for 30 s. Each mouse completed 4 trials per day with a 15-min intertrial interval. Mice were tested in groups of four consisting of a male and female WT and Irp2^−/−^ mouse until all mice completed all four trials. Training was repeated on Days 2–5.

#### 2.9.3 Acquisition probe (Day 6)

The escape box was removed. Mice were placed under a container for 30 s and then released to explore the maze for 90 s. Their navigation around the maze provided an index of how much the animal remembered the location of the escape box. Only one trial was completed by each mouse.

#### 2.9.4 Reversal learning training (Day 7–9)

The escape box was moved 180° and animals were required to adapt to a change in the location of the escape box and relearn its new position. Training was repeated on Days 8–9. Fewer training days were required for reversal learning because the location cues remained the same, and the mice were familiar with the rules of how to solve the maze.

#### 2.9.5 Reversal probe (Day 10)

Similar to the acquisition probe, the escape box was removed and the mice were given a single 90 s trial. Tracking software (ANY-maze) was used to analyze behavior after all stages were complete. Primary latency, the time taken to locate (but not enter) the escape box, was determined for each training day by averaging all four trials. A two-way repeated measures ANOVA was used to compare WT and Irp2^−/−^ performance (GraphPad Prism). For the probe trials, the maze was split into 4 zones (Fig. S5). Time spent in the “target” zone containing the escape box location was used to determine if the mouse remembered the correct location of the escape box. Unpaired t-tests and Welch's t tests were used to compare WT and Irp2^−/−^ performance (GraphPad Prism).

### 2.10 Executive function: reversal learning

Executive function using the mouse touchscreen operant platform were adapted from Mar et al. (2013). Four mouse touchscreen chambers (Lafayette Instruments; working area: 241.4 sq. cm, 230 mm in height) were housed inside sound attenuation cubicles (SAC; attenuation approx. 35 dB), each running ABET II Touch software on a Whisker Server-based Controller. A five-hole mask was used for pretraining and the extinction task, while a two-hole mask was used for correction trial training, acquisition of the visual stimulus discrimination, and reversal learning task.

#### 2.10.1 Rodent handling, hindlimb clasping, and food control

Age (2–3 months old) and sex-matched WT (n = 9) and Irp2^−/−^ (n = 13) mice were first handled and weighed for three days to calculate their free-feeding body weight. During this time, the mice were also scored for hindlimb clasping according to Guyenet et al. (2010). Briefly, each mouse was grasped by the base of the tail and lifted for 10 s on two consecutive days. Two observers, blind to experimental conditions, scored the degree of hindlimb retraction between 0 and 3. The final score was the average of both observers scores on the two days. Mice were then given 2–3g of food per 25–35g of body weight (approx. ½ pellet of rodent chow) per day to slowly reduce them to 85% of their free-feeding weight over five days. Animals were on a food-controlled diet for the duration of the experiment. Mice were group housed and weighed daily. Animals were tested 5–7 days per week. Mice progressed individually through the following stages.

#### 2.10.2 Habituation

Mice were then habituated to the testing apparatus; each mouse was placed in an operant chamber for 30 min with all the equipment on. Overnight the water in their home cages was replaced with the liquid reward (a “milkshake” consisting of 10% sweetened condensed milk) to habituate the animals to the reward. Normal water was returned on the following day.

#### 2.10.3 Pretraining

##### 2.10.3.1 Training to retrieve liquid reward

Mice were placed within the operant chamber for 30 min. To train the mice to retrieve the liquid reward from the receptacle, at the start of a trial the reward magazine light would turn on and an audible click cue would play. Mouse nose poke into the magazine would release 0.2 mL of liquid reward. An intertrial interval (ITI) of 30 s followed. Criterion for advancement was completion of at least 10 trials for two consecutive days.

##### 2.10.3.2 Training to touch the screen

Mice were then trained to touch a stimulus on the touchscreen to retrieve a reward. A small amount of 100% sweetened condensed milk was rubbed onto the touchscreen in the center response window of a five-hole mask to encourage the mice to investigate the touchscreen. At the start of the trial, a square stimulus was presented in the center response window. If untouched, the stimulus disappeared after 30 s and the magazine light was illuminated until the reward was collected. Correct nose poke touches to the stimulus were rewarded with larger volume (triple the original volume) coinciding with stimulus offset and an audio click sound. A 5 s ITI followed reward collection. Criterion was set to 30 completed trials in 60 min.

##### 2.10.3.3 Training to touch the stimulus

Mice were then trained to only touch the stimulus to receive a reward. The trial proceeded the same as in the previous step except that the mouse was required to touch the stimulus to cause a regular reward delivery, stimulus offset, and activation of the magazine light and auditory click. Advancement to the next stage required completion of 30 trials in 60-min on two consecutive days.

#### 2.10.4 Extinction task

##### 2.10.4.1 Training to initiate trial

Mice were then trained to initiate the trial by nose poke into the reward magazine. This was considered the “acquisition” phase of the extinction task. The session begins with magazine illumination and a “free” reward. Once the mouse removes its nose from the magazine the stimulus is presented on the screen. Initiation is also required after the ITI. Sessions lasted for 30 min or until the completion of 30 trials. The criterion for advancement was completion of all trials in 12.5 min over five consecutive sessions.

##### 2.10.4.2 Extinction

Each trial started with a 10-s ITI before stimulus presentation. The mouse was not required to initiate the trial. The stimulus turned off after 10 s or if the mouse touched the stimulus. A 10 s ITI started leading to the next trial. No rewards or audible clicks were delivered during this phase. The session terminated after 30 trials or 10 min had elapsed. The extinction criterion was two consecutive sessions with ≧77% omissions (23 out of 30 trials).

##### 2.10.4.3 Recovery

Recovery from extinction was accomplished by returning the mice to the acquisition program and returning reward and audible click delivery. The criterion for advancement was completion of 30 trials in 30 min for two consecutive sessions.

##### 2.10.4.4 Data analysis

An unpaired *t*-test was used to compare the number of sessions required to reach criterion between WT and Irp2−/− mice for both the acquisition and extinction phases of the task.

#### 2.10.5 Visual discrimination and reversal learning

##### 2.10.5.1 Correction trial training

A square stimulus was presented pseudorandomly in the two response windows with the limit that the stimulus wouldn't appear in the same response window more than twice in a row. An incorrect response led to stimulus offset and a 5 s “time out” period followed by a 10 s ITI. The next trial was repeated to help correct the animal's error. In correction trials, the stimulus configuration remained in the same position until the mouse corrected its error. Correction trials did not count toward the total trial limit. Sessions lasted 35 min or until completion of 30 trials, whichever came first. Criterion was set at ≧75% correct not including correction trials within 35 min for two consecutive sessions.

##### 2.10.5.2 Acquisition of the visual discrimination

The program used for visual discrimination acquisition was identical to the correction training program except two visual stimuli were presented one of which would be rewarded (CS^+^) and one would not (CS^−^). The ITI was 20 s. The left and right positions of the stimuli were determined pseudorandomly. Stimulus-reward contingencies were counterbalanced. Sessions were limited initially to 15 trials in 60 min until the subject could complete all trials in 30 min. These trials were binned into full 30-trial sessions for analysis. If a mouse did not complete the full 15 trials, the remaining trials would be added to the next session. The criterion for this stage was completion of 30 trials in 60 min with ≧85% accuracy excluding correction trials.

##### 2.10.5.3 Reversal learning

At the start of reversal learning the reward contingencies for each subject were reversed; the CS^+^ became the CS^−^, and vice versa. Mice were therefore required to reverse their response to earn a reward. Sessions were again initially limited to 15 trials in the same manner as previously described. Criterion was completion of 30 trials in 60 min with ≧85% accuracy excluding correction trials.

##### 2.10.5.4 Data analysis

Data were subjected to RM ANOVA with a Šídák correction for multiple comparisons (GraphPad Prism). When data sets significantly violated the homogeneity requirement for a repeated measures design, the Geisser-Greenhouse was used to calculate a more conservative p value for each F ratio. An unpaired *t*-test with a Welch correction was used to compare new learning errors due to a significant difference in variances (p < 0.01).

## 3 Results

### 3.1 Irp2-null mice show a robust motor deficit

**Fig. 1:**
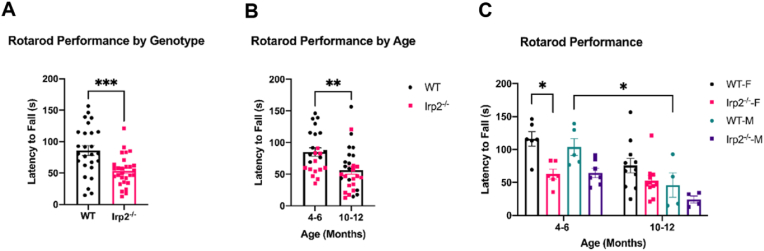
Irp2-null mice show motor impairments on the rotarod task A) Irp2^−/−^ mice fall off the accelerating rotarod faster than WT mice. B-C) Rotarod performance also depends on age and sex. *p < 0.05; **p < 0.01; ***p < 0.00.

To determine if the newly backcrossed Irp2^−/−^ mice have a similar motor deficit as the original Rouault model, two motor tests were performed. On an accelerating rotarod test Irp2^−/−^ mice had a nearly 40% reduction in latency to fall (Fig. 1A; t = 3.579, df = 40.82, p < 0.05). Rotarod performance also depended on age (Fig. 1B; t = 3.15, df = 52, p < 0.05). Previous work (LaVaute et al., 2001; Jeong et al., 2011) has described the neurodegenerative disease phenotype of Irp2^−/−^ mice as progressive, making it difficult to compare results from different groups using mice of different ages and/or excluding female mice in their experiments (Galy et al., 2006; Zumbrennen-Bullough et al., 2014). To investigate how age and sex affect Irp2^−/−^ rotarod performance, our cohort included male and female mice from two age groups: a 4–6-month-old group (‘Young’) and 10–12-month-old group (‘Middle’). Ideally this comprehensive approach would reveal an appropriate “window” for rotarod testing in Irp2^−/−^ mice. Further three-way ANOVA analysis revealed a significant effect of age (Fage (1,46) = 21.09, p < 0.05) accounting for approximately 23.62% of variation as well as a significant (Fsex (1,46) = 18.14, p < 0.05) effect of sex (5% of variation). Similarly, to the previous analysis, the effect of genotype was significant (Fgenotype (1,46) = 4.497, p < 0.05) and accounted for 20.31% of variation.

No interaction effects were significant. Interestingly, the greatest difference between WT and Irp2^−/−^ mice was in young, female mice, the group most often excluded in other studies (Fig. 1C). The use of older males for evaluation of motor defects may have reduced the ability to properly detect motor compromise in previous studies (LaVaute et al., 2001; Galy et al., 2006; Zumbrennen-Bullough et al., 2014). An older (>12-month-old group) could not be assembled to be included in this study, but due to the negative effect of age on WT rotarod performance, a difference between genotypes is unlikely. Because lab mice increase in weight over time and male mice typically weigh more than female mice (Fig. S2), we hypothesized that differences in weight could be the underlying factor that accounted for the effect of age and sex on rotarod performance. To address this possibility, the data was analyzed again with an ANCOVA model using weight as the covariate. A between-subjects effect of weight on rotarod performance was discovered (Fweight (1,45), p < 0.05)) validating the need to control for this factor. The effect of genotype was again significant (Fgenotype (1,45) = 6.884, p < 0.05), but both sex (Fsex (1,45) = 0.002, ns)) and age (Fage (1,45) = 2.184, ns) were insignificant (Fig. S1). The mean difference in estimated marginal means between WT and Irp2^−/−^ mice was 40.4 s (weight = 33.26g).

**Fig. 2:**
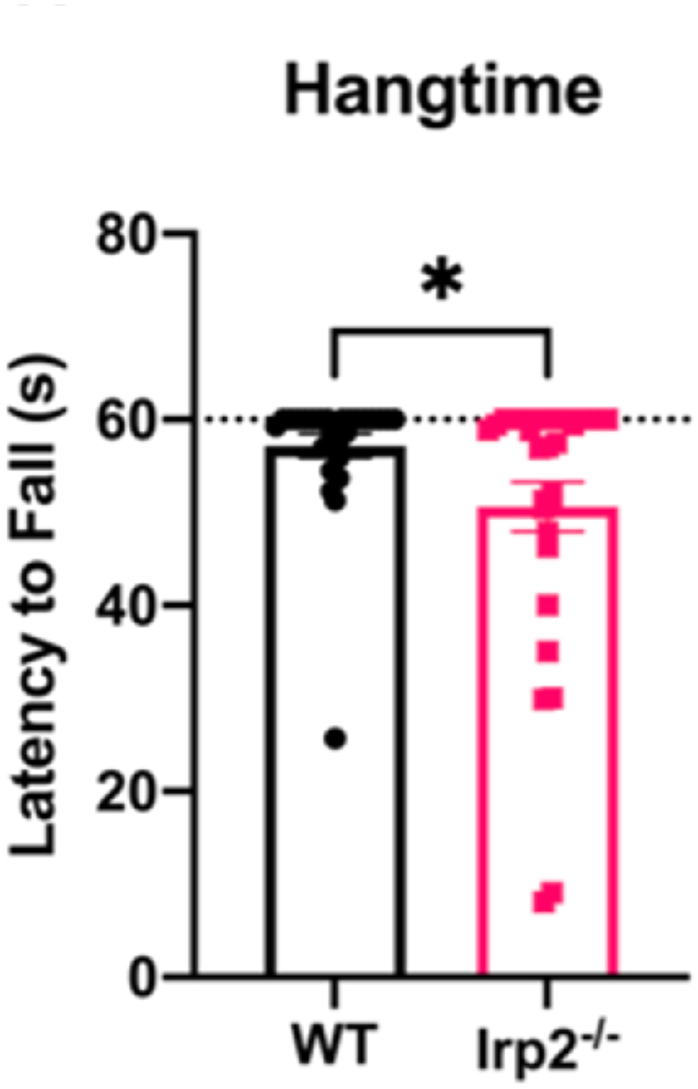
Irp2^−/−^ mice show a reduction in hangtime test performance Irp2^−/−^ mice show a reduction in latency to fall during the hangtime test. Dotted line represents endpoint of test * p < 0.05.

These results indicate that age and sex per se do not influence rotarod performance, but underlying differences in weight likely contribute to the appearance of an effect in raw data. This highlights the need for careful selection of WT and Irp2^−/−^ mouse cohorts including appropriate age groups of male and female mice. Previous work in our lab utilized a hangtime test to assess motor function/grip strength (LaVaute et al., 2001). In this test, mice are placed on a wire cage/grid that is flipped upside down for 60 s. Unlike the data for the rotarod test, age could not be used as a predictive factor because of unequal variances across age groups. We applied the ANCOVA model, as with the rotarod analysis, with weight as the covariate. This revealed a lower weight in the Irp2^−/−^ mice (Fweight (1,52) = 14.438, p < 0.05; Fig. S2) causing the difference between the groups in their latency to fall during the hangtime test (F_genotype_ (1,52) = 14.438, p < 0.05; Fig. 2). Previous studies have tested older, and likely overweight male mice, primarily because the Irp2−/− phenotype was previously described as progressive. Our findings suggest that only young (6 months or less) male and female mice should be used in motor tests of Irp2^−/−^ models. Although the compromise was less apparent on the hangtime test for grip strength, which was most likely due to limiting the test to 60 s, the combination of both motor tests demonstrates that loss of Irp2 results in a deficit in motor control.

### 3.2 Irp2-null mice show an increase in nociceptive thermal tolerance

**Fig. 3:**
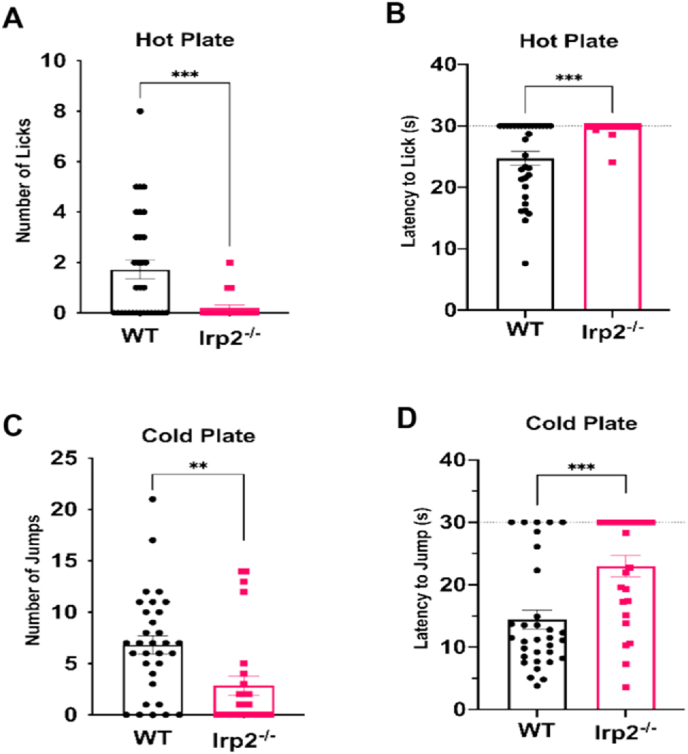
Irp2-null mice exhibit a deficit in somatosensory perception WT and Irp2^−/−^ mice tested on a hot (A–B) and cold (C–D) plate assays. Irp2^−/−^ mice had a significant decrease in number of hind-paw licks (A) and increase in latency to first hind-paw lick (B). Irp2^−/−^ mice had a significant decrease in the number of jumps (C) and a significant increase in latency to first jump (D). In the absence of an event, latency was recorded at 30 s (dotted line). **p < 0.01, ***p < 0.001.

The Leibold group reported an increase in nociceptive thermal tolerance in their Irp2^−/−^ mouse model using a hot plate assay (Zumbrennen-Bullough et al., 2014). To investigate and expand on this phenotype, both hot (52 °C) and cold (0 °C) plate assays were performed. Age and sex-matched WT and Irp2^−/−^ mice were exposed to each thermal stimulus for 30 s. The number of hind-paw licks (hot) and jumps (cold) were recorded along with the latency to the first event (latency of 30 s was used in the absence of the event). For both stimuli, Irp2^−/−^ mice had a significant reduction in thermal nociceptive sensitivity as evidenced by a decrease in the number and an increase in latency of nocifensive behaviors (Fig. 3).

### 3.3 Irp2-null mice show an increase in nociceptive thermal tolerance

The Leibold group reported an increase in nociceptive thermal tolerance in their Irp2^−/−^ mouse model using a hot plate assay (Zumbrennen-Bullough et al., 2014). To investigate and expand on this phenotype, both hot (52 °C) and cold (0 °C) plate assays were performed. Age and sex-matched WT and Irp2^−/−^ mice were exposed to each thermal stimulus for 30 s. The number of hind-paw licks (hot) and jumps (cold) were recorded along with the latency to the first event (latency of 30 s was used in the absence of the event).

For both stimuli, Irp2^−/−^ mice had a significant reduction in thermal nociceptive sensitivity as evidenced by a decrease in the number and an increase in latency of nocifensive behaviors (Fig. 3). Specifically, on the hot plate, WT mice licked more frequently (t = 3.879, df = 36.6, p < 0.05) and more quickly (t = 4.366, df = 33.68, p < 0.05). Similarly, on the cold plate, WT mice jumped more frequently (t = 3.082, df = 56, p < 0.05) and more quickly (t = 3.745, df = 56, p < 0.05).

Previous work has looked at the effect of Irp2 ablation in lower motor neurons (Jeong et al., 2011), but these results indicate that a similar effect of functional iron deficiency might be observed in peripheral sensory neurons resulting in a somatosensory defect in Irp2^−/−^ mice. It is also possible that a supraspinal mechanism is responsible since hot plate assays are also able to detect sensorimotor defects after spinal cord injury (Giglio et al., 2006; Bannister and Dickenson, 2020).

### 3.4 Irp2-null mice are mildly active in an open field test of spontaneous locomotion

**Fig. 4:**
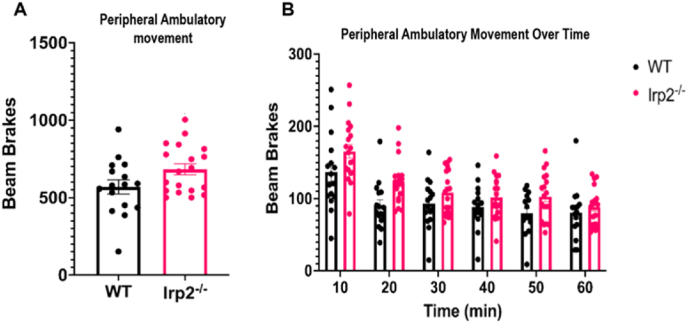
Irp2-null mice show a mild increase in ambulatory movement WT and Irp2^−/−^ mice completed three 60-min trials in an open field. A) Irp2^−/−^ mice have an increase in average periphery ambulatory movement B) which is most evident in the first 20-min of the trial.

Previous work with Irp2^−/−^ mice has produced conflicting results for the effect of IRP2 ablation on spontaneous locomotion (Galy et al., 2006; Zumbrennen-Bullough et al., 2014). It was important to establish if horizontal movement in our Irp2^−/−^ model is reduced enough to hinder performance in future behavioral tests. A PAS-open field system was used to track mouse locomotion over three, 60-min trials. Age and sex-matched Irp2^−/−^ mice showed a trend (t = 1.982, df = 33, p = 0.056) toward increased ambulatory activity in the periphery of the open field (Fig. 4A). This agrees with results reported in studies of the Hentze Irp2^−/−^ model (Galy et al., 2006). No differences were observed in fine movement or center ambulatory movement (Fig. S3). Periphery ambulatory movement beam brakes were binned into 10-min intervals to look at differences in movement over time. A two-way RM ANOVA revealed a significant effect of time (F_time_ (5, 85.9) = 2.603, p < 0.05) with movement declining over time as the mice habituate to the apparatus. The Irp2 ablated mice were mildly active relative to the WT (F_genotype_ (1, 33) = 4.622, p < 0.05) but there was no interaction (F_genotype__x__time_ (5, 165) = 1.748 ns; Fig. 4B).

### 3.5 Loss of Irp2 does not affect anxiety-like behavior

**Fig. 5:**
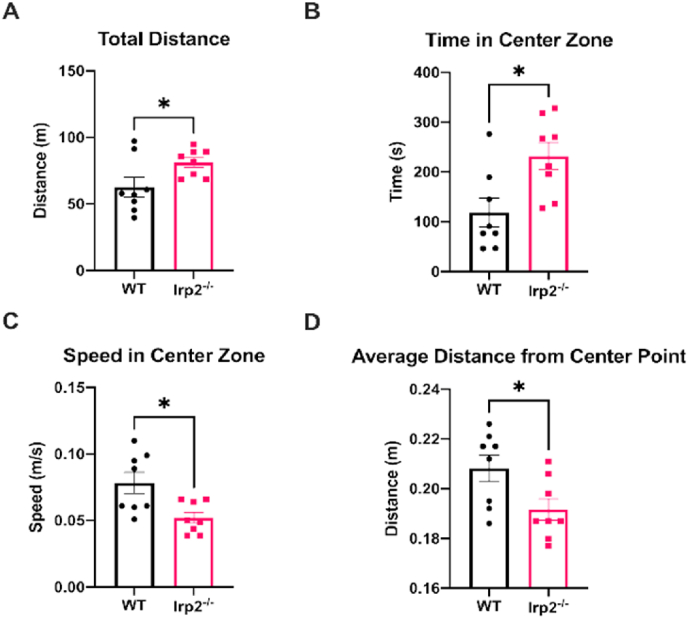
Irp2^−/−^ mice are not anxious in the open field In a single trial of the square open field test, Irp2^−/−^ travel greater distances and show a reduction in thigmotactic behavior. A) Overall distance travelled increases in Irp2^−/−^ mice while B) time spent in the center zone also increased. C) Speed in the center zone and average distance from the center point decreased in Irp2^−/−^ mice. An unpaired *t*-test was used to compare WT and Irp2^−/−^ performance * p < 0.05.

**Fig. 6:**
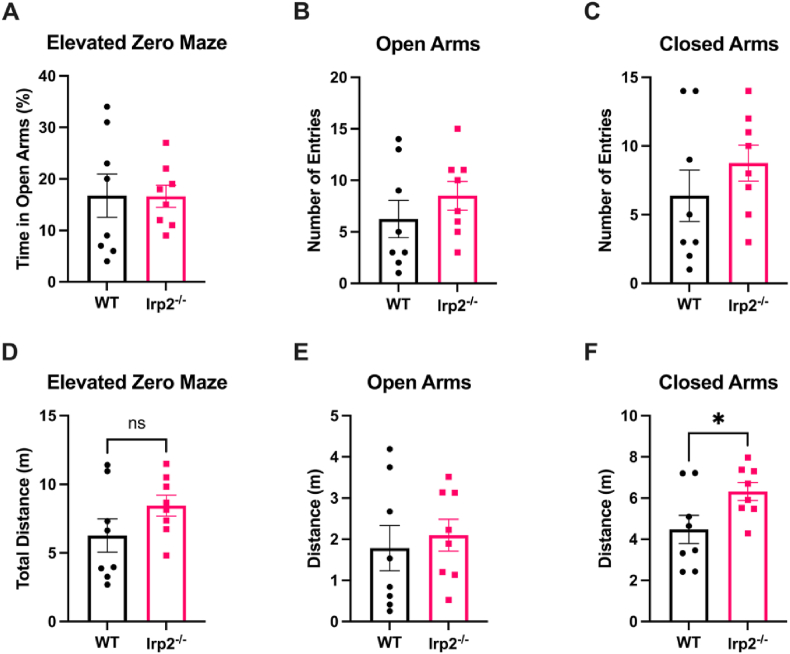
Irp2^−/−^ mice are not anxious in the elevated zero maze In an elevated zero maze test, Irp2^−/−^ mice spend an equal amount of time in the open arms of the maze (A) entered open arms (B) and closed arms as much as controls (C) travelled similar distances as WT (D, E), except in closed arm (F). These data indicate that anxiety is unaffected by loss of Irp2 *p < 0.05.

Based on previous work investigating iron metabolism and anxiety (Patel et al., 2002; Texel et al., 2012; Vroegindeweij et al., 2019; Wang et al., 2019), we hypothesized that the mild increase in activity could be due to an increase in anxiety in the Irp2^−/−^ mice. Thigmotaxis is the tendency of mice to stay close to walls in an open field space, and an increase in thigmotaxis is a well-accepted marker of anxiety (Simon et al., 1994; Wang et al., 2019; Powell et al., 2022). It is also possible that the increase in ambulatory movement is due to hyperactivity alone. To address this issue, a second open field test was conducted, this time using a larger, square open field. The center zone of this larger open field was a more aversive environment (due to the greater distance between the center and the walls of the apparatus) than in the previous test. An increase in thigmotaxis in Irp2^−/−^ mice, would be exacerbated in this apparatus. Additionally, only a single, 30-min trial was completed since the mice habituate to the environment over repeat trials. Irp2^−/−^ mice exhibited an increase in locomotion with a nearly 30% increase in total distance travelled (t = 2.274, df = 14, p < 0.05; Fig. 5A). Surprisingly Irp2^−/−^ mice spent nearly twice as much time in the center zone (t = 2.876, df = 14, p < 0.05), had a lower average speed in the center zone (t = 2.929, df = 14, p < 0.05), and on average located themselves more closely to the center point of the apparatus than WT mice (t = 2.433, df = 14, p < 0.05; Fig. 5B–D). There was also a tendency for the Irp2^−/−^ mice to spend more time in the center zone on each visit (t = 2.001, df = 14, p = 0.064) and visit the center zone more often (t = 2.093, df = 14, p = 0.055; Fig. S4). Thus, if anything, these metrics point to low activity and a general reduction in anxiety in the Irp2 ablated mice (see Fig. 6).

Another commonly used test for anxiety in rodents is the elevated-zero maze (EZM) which works similarly to the elevated-plus maze (EPM) but lacks a center region (Braun et al., 2011; Tucker and McCabe, 2017). The EZM and EPM consist of an elevated open platform with “closed” wall-containing sections and “open” exposed sections. Anxious mice typically spend more time in the closed sections of the maze, so if loss of Irp2 is anxiolytic, then Irp2^−/−^ mice would spend more time in the open sections of the maze. This was not the case in a single, 5-min trial. Although the Irp2^−/−^ mice moved more in the closed arms (p < 0.05) thereby traveling slightly greater

distances, these animals spent as much time in the open sections of the maze as WT mice (t = 0.053, df = 14, ns) confirming the general lack of anxiety in mice with Irp2 ablations.

### 3.6 Working memory in Irp2^−/−^ mice is intact

**Fig. 7:**
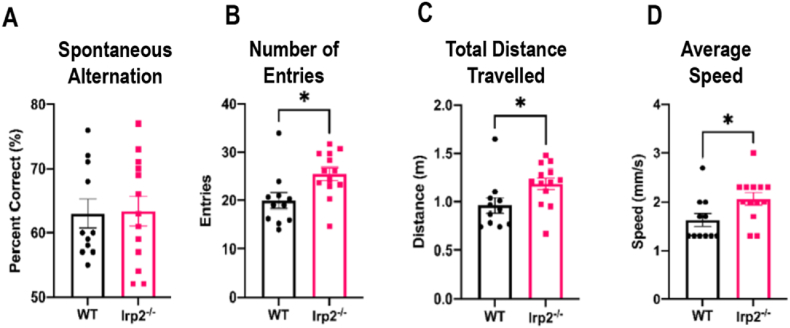
Spatial working memory is intact in Irp2-null mice Spatial working memory in Irp2^−/−^ mice was assessed using a Y-Maze test. A) There was no significant difference in spontaneous alternation indicating that working memory is intact. Irp2^−/−^ mice however showed a general increase in motor activity evidenced by an increase in B) total number of entries, C) distance travelled, and D) average speed of movement. *p < 0.05.

Working memory is the ability to temporarily hold and manipulate information. To assess whether working memory is affected by Irp2 ablation, a simple Y-maze test was used. When placed in a Y-maze, mice tend to explore each arm individually before returning to a previously explored arm (Kraeuter et al., 2019; Jaafari suha et al., 2022); this spontaneous alternation requires the use of working memory so as not to revisit previously-explored locations. This behavior requires interaction between different regions of the brain including the hippocampus and prefrontal cortex (Swonger and Rech, 1972; Lalonde, 2002; Liu et al., 2018). The WT mice performed as expected with a spontaneous alternation percentage greater than chance. Irp2^−/−^ mice did not significantly differ from WT mice indicating that spatial working memory was intact (Fig. 7A) but they did show deficits indicative of general motor problems. For example, they made many entries into the arms of the maze (t = 2.67, df = 22, p < 0.05; Fig. 7B), travelled longer distances (t = 2.28, df = 22, p < 0.05; Fig. 7C) and moved with greater speed (t = 2.33, df = 22, p < 0.05; Fig. 7D).

### 3.7 Irp2-null mice show poor navigation strategy

Due to the known association between dietary iron deficiency, learning and memory, and hippocampal function (Weinberg et al., 1979; McEchron et al., 2005; Ennis et al., 2019; McCann et al., 2020; Porras and Rouault, 2022), we hypothesized that disruption of iron metabolism regulation by ablation of Irp2 could impair long-term learning and memory in a similar manner. A Barnes maze test was used to assess long-term spatial learning and memory in our Irp2^−/−^ mice. Originally developed in 1979 for rats and then later adapted for mice, this “dry” version of the Morris water maze (MWM) is less stressful and diminishes possible complications that arise from the Irp2^−/−^ motor phenotype. Like the MWM, the Barnes maze is a hippocampal-dependent task in which mice learn the location of a fixed escape location using distal visual cues (Barnes, 1979; Morris, 1984; Bach et al., 1995; Harrison et al., 2009; Van Den Herrewegen et al., 2019). Additional training stages allowed for the assessment of cognitive flexibility i.e., the ability to rapidly change behavior due to changing circumstances (Izquierdo et al., 2017). In this case the changing circumstance was the movement of the escape box 180° from the initial target hole.

**Fig. 8:**
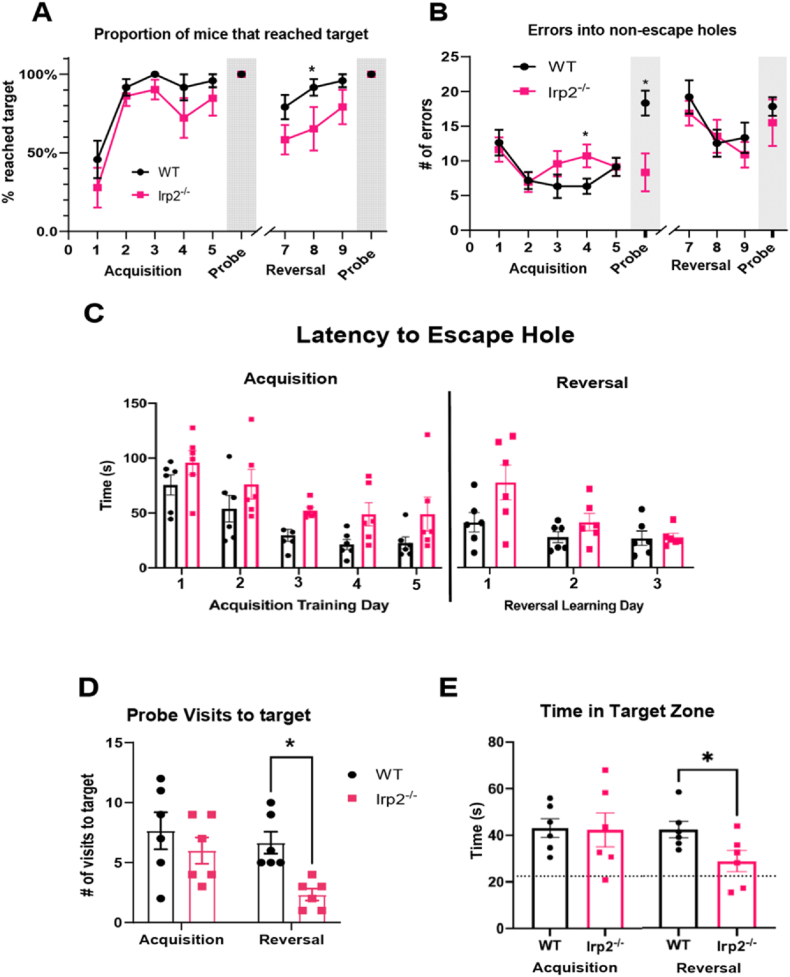
Irp2 ablation impairs search strategy WT and Irp2^−/−^ mice performance during a Barnes maze task. Training days consisted of four 3-min trials. 90-second probe trials were conducted 24hrs after the last training day. A) proportion of mice able to locate the escape hole, B) mean errors defined as entries into non-escape holes, C) latencies to escape hole (also known as primary latency), D) number of visits to the escape hole, and E) time spent in the target zone. Dotted line in E represents the expected time in target zone due to random chance. *p < 0.05.

During acquisition, animals from both groups improved over training to reach the goal on almost all trials (Fig. 8A; Fday (2.425, 24.25) = 21.36, p < 0.05) and reduced the number of errors into the non-escape holes during learning (Fig. 8B; Fday (2.447,24.47) = 4.246, p < 0.05). Their latency to reach the escape platform (primary latency) became faster with increasing training day (Fday (2.057, 20.57) = 13.0, p < 0.05) accounting for 38.5% of the total variation. A significant genotype effect (Fgenotype (1,10) = 7.605, p < 0.05)was due to the Irp2^−/−^ mice taking longer than the WT controls in locating the escape hole, accounting for 13.76% of total variation (Fig. 8C). When the escape hole was removed during the acquisition probe, to assess their memory for the location of the escape hole, the WT mice made many entries into non-escape holes in their search for the absent escape hole. This search strategy was reduced in the Irp2 ablated mice (t = 4.589, df = 15, p < 0.05; Fig. 8B). Irp2 ablated mice made a similar number of visits to the target zone (Fig. 8D) and spent a comparable duration of time as the WT in the target zone (Fig. 8E). Other aspects of performance such as distance travelled, and path efficiency did not differ between the groups during acquisition (Fig. S6).

We then challenged the animals by moving the escape platform to a location 180^o^ from its original location. In other words, the escape hole was now in the opposite location so mice were required to relearn the location of a new escape hole and adapt to this new environmental change. In this new context, all animals irrespective of group eventually found the new escape hole with increasing session time (F_day_ (1.55, 15.5) = 11.28, p < 0.05)). However, across the 3-day reversal training period, Irp2 ablated mice found the new location less frequently than did WT mice F_genotype_ (1,10) = 5.930, p < 0.05); Fig. 8A). Notably, this deficit was not due to the increased number of non-escape hole errors while searching for the new platform since this did not differ between the two groups (t_wt- Irp2−/−_ = 0.695, df = 8.66 ns; Fig. 8B). The latency to find the escape hole was reduced with increasing training day for all animals (Fday (1.55, 15.5) = 11.28 p < 0.05), but no effect of genotype (Fgenotype (1, 10) = 3.289 ns) or interaction (Fgenotype x day (2,20) = 3.216, ns) was observed.

During the reversal probe trials, when the escape platform was removed from the new reversed location, the Irp2^−/−^ mice made fewer visits to the new target hole (F_genotype_ (1,10) = 5.226, p < 0.05). The, Irp2^−/−^ mice also spent less time in the target zone relative to WT controls (t_wt- Irp2−/−_ = 2.352, df = 10, p < 0.05). On average, the Irp2^−/−^ mice spent 28.97s (±4.54 SEM) in the target zone which is only slightly above chance (22.5 s). In contrast, the WT mice spent 42.67s (±3.65 SEM) in the target zone, essentially the same as their acquisition probe which was 43.12 s (±4.08 SEM) suggesting that Irp2−/− mice were less exploratory.

**Fig. 9:**
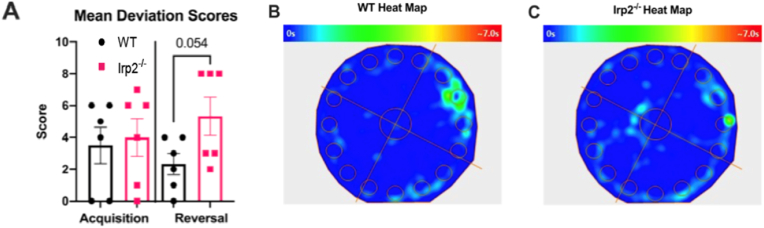
Irp2^−/−^ mice tend to have a higher deviation score than WT mice A) Mean deviation scores of WT and Irp2^−/−^ mice during the acquisition and reversal learning probe. B-C) Heat maps of reversal learning probe location of B) WT and C) Irp2^−/−^ mice.

We then examined the deviation score for each mouse which was determined based on the number of holes between the first hole they visited in the outer-most layer to the target hole/escape box location on both the acquisition and reversal probes. A score of 0 corresponded to a mouse visiting the target hole first while a score of 8 corresponded to the hole 180° from the target hole (Fig. S5). On the reversal probe a score of 8 would indicate the mouse visited the initial target hole first. The groups did not differ during the acquisition probe, but during the reversal learning probe (Fig. 9A), the deviation score, in the Irp2 null mice trended higher than WT controls (t_wt- Irp2−/−_ = 2.183, df = 10, p = 0.054) suggesting a difficulty in the Irp2 group in moving away from the initial target location. The heat maps in Fig. 9B and C illustrate the difference in behavior of WT and Irp2^−/−^ mice during the acquisition and reversal probe. In addition, Irp2^−/−^ mice showed reduced path efficiency - the distance travelled to the first visit to the target hole divided by length of the shortest, most direct path possible, suggesting an impaired search strategy (Fig. S6).

Taken together, the evidence suggests that the loss of Irp2 compromised the animals’ searching strategy, which made it difficult for these animals to adapt to an environmental change.

### 3.8 Irp2-null mice are impaired in frontal-executive function

Executive function refers to a series of higher order processes that contribute to flexible, goal-directed behavior which governs more basic cognitive properties. These processes include directing attention, risk-related decision-making, and response inhibition (Jurado and Rosselli, 2007; Diamond, 2013; Orsini et al., 2015), and rely on an intact prefrontal cortex (Friedman and Robbins, 2022). Deficits in executive function contribute to a variety of psychiatric and neurological disorders (Elliott, 2003; Holmes and Wellman, 2009). Since MRIs of IRP2-null patients demonstrate frontal lobe disruption (Porras and Rouault, 2022), frontal-executive function could be affected by loss of Irp2 in mice.

Various methods have been developed to investigate different aspects of executive function in animal models (Chadman et al., 2009; Chudasama, 2011). To examine possible executive function defects in Irp2^−/−^ mice, we adapted a protocol from Mar et al. (2013) which uses the touchscreen operant chamber platform. We decided to focus on two aspects of executive function: extinction i.e., the ability to stop making a response once it is no longer rewarded, and reversal learning. In contrast to the Barnes maze which requires spatial navigation in an open aversive environment, the stimulus discrimination and reversal learning task is appetitive, visual, and limits the mouse to only two computer graphic choices that occur to the left and right of a touchscreen. Thus, the animal must be cognitively engaged in making discriminating stimulus features and targeted in its response. Moreover, discrimination and reversal learning tasks using touchscreens is a commonly used test in human patients with frontal pathology (e.g., Fellows and Farah, 2003) to detect impairments in cognitive flexibility, so it makes sense to use this task for its translational application.

A cohort of very young (2–3 months old) sex-matched WT (n = 9) and Irp2^−/−^ (n = 13) mice were trained to respond to two visual stimuli on the touchscreen to receive a “milkshake” reward. The WT and Irp2^−/−^ mice were first scored for degree of hindlimb clasping as a qualitative assessment of the presence and extent of the Irp2 neurodegenerative phenotype in the young cohort of mice used for the executive function tasks. We found that that Irp2^−/−^ mice retracted their hindlimb significantly (t_wt- Irp2−/−_ = 2.728, df = 23, p < 0.05; Fig. S7). Both WT and Irp2^−/−^ mice then progressed through the various pretraining stages at essentially the same rate (Fig. S8).

**Fig. 10:**
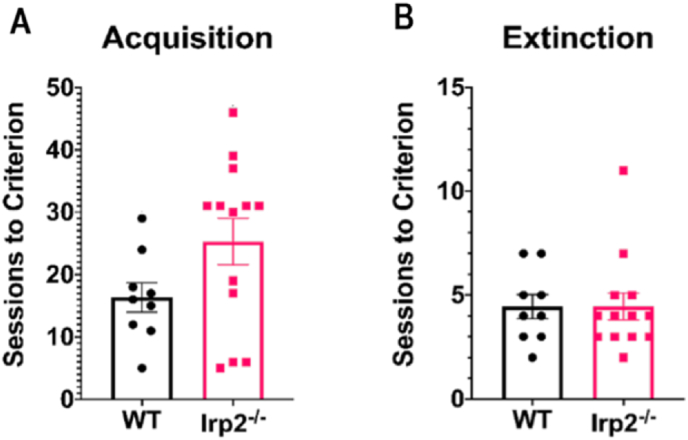
Irp2^−/−^ mice readily extinguish a previously learned non-rewarded response WT and Irp2^−/−^ mice completed an extinction task. A) number of sessions to reach criterion during acquisition, B) number of sessions to reach extinction criterion. The groups did not differ at either stage (p < 0.05).

For the extinction task, mice were first required to initiate the trial by making a nose poke into the reward magazine, touch the stimulus in the center response window and collect the reward. The criterion for this stage was completion of 30 trials in 12.5 min on five consecutive sessions. Overall, there were no real difference between the groups in the number of sessions to reach criterion performance (t_wt- Irp2−/−_ = 1.825, df = 20, p = 0.083; Fig. 10A) During the extinction, no reward or conditioned reinforcers (audible clicks) were provided after touching the stimulus. Both WT and Irp2^−/−^ extinguished the behavior in an average of four sessions (23 or more omissions in 30 trials over two consecutive sessions; Fig. 10B). Thus, the Irp2^−/−^ mice were able to adapt their behavior and extinguish their response when it no longer elicited a reward.

In the visual discrimination and reversal learning task, mice were initially required to discriminate two visual stimuli presented on a touchscreen, of which only one was positively associated with reward, and then reverse their response when the stimulus-reward contingencies were reversed. We first examined the total number of trials required to reach criterion levels of performance for both acquisition and reversal in a repeated measures analysis. This revealed that although the Irp2^−/−^ mice needed more trials (F_genotype_ (1,19) = 6.571, p < 0.05), this difference was largely accounted for by the Irp2^−/−^ mice requiring more trials before hitting criterion in the reversal stage (t_wt- Irp2−/−_ = 2.610, df = 38, p < 0.05). We further examined the type of errors committed during testing. Specifically, when an animal made an error, the same trial with the same right/left stimulus configuration was repeated as a correction trial, until the animal made a correct response. Therefore, correction trials errors failed to dissociate repeated responses made to the visual stimulus from repeated responses on the side on which it was presented. Once the animal corrected its mistake, it received a non-correction trial, in which the left/right stimulus configuration was spatially opposite or the same, presented in a pseudorandom manner. Therefore, a non-correction trial error was a perseverative response directed to the previously rewarded stimulus.

**Fig. 11:**
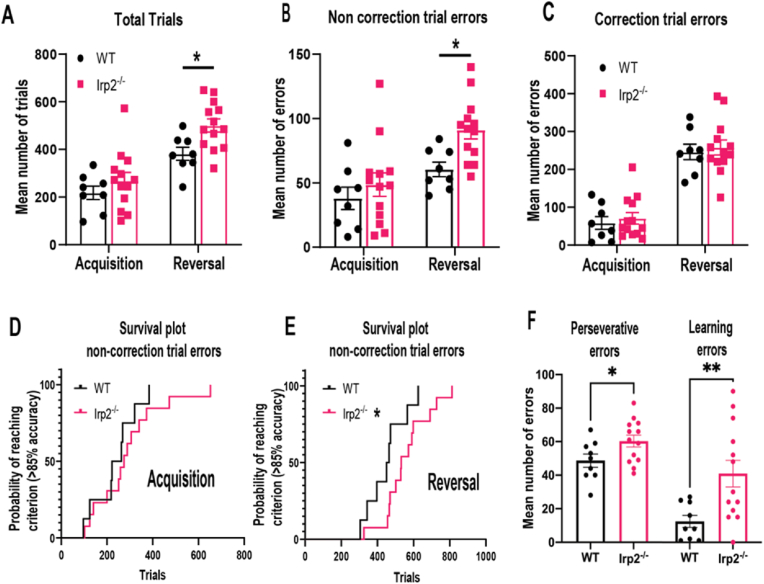
Irp2^−/−^ mice are cognitively inflexible WT and Irp2^−/−^ completed a reversal learning task using the touchscreen operant platform. A) Both WT and Irp2^−/−^ mice need more trials to reach criterion levels of performance. B) During reversal, Irp2^−/−^ mice made many non-correction trial errors. C) Irp2^−/−^ mice needed as many repeat correction trials just like WT to correct their mistakes. D, E) Non correction trials were grouped into 100 trial bins to generate survival plots for both genotypes. The survival plot for the Irp2^−/−^ mice were slower to acquire the reversal only. F) During reversal, Irp2^−/−^ mice make many stimulus-perseverative errors but also many learning errors. *p < 0.05.

We subjected the data to a repeated measures analysis and discovered that the Irp2^−/−^ mice were impaired at the reversal stage committing many non-correction trial errors (F_genotype_ (1,19) = 13.41, p < 0.05, during reversal (t_wt- Irp2−/−_ = 2.582, df = 38, p < 0.05) because they were unable to suppress their responding to the previously rewarded stimulus (Fig. 11B and C). We computed survival curves for both acquisition and reversal to establish the likelihood of animals reaching criterion performance after a certain number of trials (Fig. 11D and E). This confirmed that relative to the WT, the Irp2^−/−^ mice were indeed slower to reach criterion performance (χ^2^_reversal_ = 4.03, df = 1, p < 0.05), which was again, specific to reversal (χ^2^_acquisition_ = 0.589, df = 1, ns).

During reversal learning, the number of non-correction trial errors were further analyzed according to two learning stages (Jones and Mishkin, 1972; Dias et al., 1996; Chudasama and Robbins, 2003). Errors made before significantly below chance-level performance (40% correct for 30 trials), were classified as ‘stimulus-perseverative.’ Errors committed between 40% and 85% correct performance suggested the animal made a response away from the previously rewarded stimulus and continued to respond randomly until they shifted to the stimulus positively correlated with reward. These errors were classified as 'learning errors.' Fig. 11F shows the Irp2^−/−^ mice made more perseverative errors (t_wt- Irp2−/−_ = 4.332, df = 20, p < 0.05) and learning errors (t_wt- Irp2−/−_ = 3.268, df = 20, p < 0.05). Interestingly, there was a greater difference in the number of errors between WT and Irp2^−/−^ mice during the “learning” stage than the “perseverative” stage (28.48 ± 8.7 vs 11.64 ± 5.4 respectively) corresponding with an approximately 3-fold increase in learning errors in Irp2^−/−^ mice. Finally, latencies to collect reward (F_genotype_ (1,19) = 3.123, ns) or make a response (F_genotype_ (1,19) = 0.418, ns) did not differ between groups. Taken together, these data confirm that Irp2 ablations cause deficits in cognitive flexibility.

## 4 Discussion

In the current study, we have attempted to broaden our understanding of the Irp2^−/−^ phenotype. By backcrossing the original model to C57Bl/6J mice, we were able to ensure that any observed differences between our Irp2^−/−^ model and those of other groups were. not due to differences in genetic background. The extensive testing on both the rotarod and hangtime tests definitively establish a motor defect in Irp2^−/−^ mice. These results also identify a “window” around 4–6 months of age when the defect is most apparent in these tests. The previously observed mildness of the motor phenotype was likely due to the exclusive use of older, male mice (LaVaute et al., 2001; Galy et al., 2006; Zumbrennen-Bullough et al., 2014).

The somatosensory deficit observed on both the hot and cold plate assay is an interesting addition to our Irp2^−/−^ model phenotype which replicates and extends previous work done with the Leibold Irp2^−/−^ model (Zumbrennen-Bullough et al., 2014). Hot and cold plate assays are very simple measures of nociception that are sensitive to confounding factors in hyperactivity and gait. In our case, however, the Irp2 ablation made mice slower to lick or jump which is opposite to what we might expect with hyperactivity. Further assays less sensitive to these confounds will help better understand which parts of the somatosensory system could be affected by loss of Irp2. It would be interesting to examine the health of peripheral sensory neurons to see if they have similar mitochondrial abnormalities and dysfunction as lower motor neurons in these mice (Jeong et al., 2011).

We have also shown in several contexts that Irp2^−/−^ mice are active in ways which likely corresponds to previously reported increases in motor activity (Galy et al., 2006). However, the motor activity deficits were not equivalent across all tasks; motor deficits were clearly evident during Y-Maze performance even though the animals were not impaired in memory, but in other maze settings, there was no evidence of increased locomotor activity. Moreover, the Irp2^−/−^ mice showed less thigmotactic behavior in the open field and elevated zero maze suggesting that the increased motor activities could not be attributed to an increase in anxiety. In combination with the fast fall latencies in the rotarod and hangtime tests, the most parsimonious explanation is that the motor deficit in the Irp2^−/−^ mice is related to an increase in maladaptive impulsive behavior and possibly novelty/sensation seeking behavior (Plagge et al., 2005; Yamashita et al., 2013; Dent and Isles, 2014). The fast fall latencies and the spontaneous over activity in the Y-Maze is consistent with this view, but the hypothesis needs to be tested directly.

The results from the Y-maze and Barnes maze indicate that both working and long-term memory is intact in Irp2^−/−^ mice. In fact, the results of the Barnes maze reversal probe indicate that Irp2^−/−^ mice can recall a specific spatial location many days after the last acquisition trial. The difference in primary latency during Barnes maze acquisition training is more difficult to interpret. Perhaps the mice are less driven to find the escape box location immediately and are more interested in exploring the maze due to their hyperactive phenotype. During subsequent training the maze would no longer be a “novel” environment, and thus by the acquisition probe Irp2^−/−^ mice performed similarly to WT mice.

Irp2^−/−^ mice had some difficulties when adjusting to a new solution when the escape platform was placed in the opposite location during the probe trial. For the most part, their performance in the reversal probe was worse than their performance in the acquisition probe. This could perhaps be due to due to the difference in number of training days (5 vs 3) before the probe trial. However, no mouse failed to visit the location of the hole even though their latency to find the escape holes decreased in Irp2^−/−^ mice. In addition, the latencies improved indicating that their learning improved. The combination of a high error rate in holes closer to the initial target hole combined with fewer visits and time spent in the new target zone suggests a failure of Irp2^−/−^ mice to switch from random/serial search strategies to a direct search strategy.

The results from the touchscreen experiments establish an executive function deficit in Irp2^−/−^ mice. It's interesting that in the extinction task, there was a slight tendency for Irp2^−/−^ mice to require more sessions to reach the acquisition criterion. The addition of trial initiation seemed to be more difficult for Irp2^−/−^ mice to learn than other pretraining steps. This could be indicative of an attention problem in these mice. They may be “distracted” from noticing the ITI has ended, and the receptacle light is on for trial initiation, leading to a longer completion time and failure to meet the criterion (five consecutive sessions with a completion time of 12.5 min or less). This hypothesis however would need to be further tested with a 5-choice serial reaction time task (5-CSRTT) to definitively make a conclusion of an attention defect. There was no difference in sessions required to reach criterion in the extinction stage though, indicating that Irp2^−/−^ mice in this context do not have a problem with extinction.

The reversal learning task demonstrated that, Irp2^−/−^ mice had a difficulty in reversing their response. Irp2^−/−^ mice made many stimulus-perseverative errors. Thus, the Irp2 ablation impacted the animals’ normal ability to adjust and adapt to changes in its immediate environment. This impairment was not a generalized perseverative deficit since the inflexibility did not occur for correction trials that occurred in the same left/right spatial positions. Nor was it impacted by motivational changes since their speed of response and reward collection latencies were in the normal range. Thus, these animals showed a degree of inflexibility that was specific to the stimulus that was previously associated with reward. It is possible that Irp2^−/−^ mice were easily distracted by the presence of the previously rewarded stimulus that made it harder to switch response. Although this result appears at odds with the results of the extinction task, we note that the stimulus-reward contingency in the extinction task is different to reversal learning. We used a deterministic reversal learning task wherein choosing the CS + stimulus results in a reward 100% of the time. Mice typically take longer to reach criterion in probabilistic reversal learning tasks, i.e., tasks where the reward probability of the CS + stimulus is less than 100% and the reward probability of the CS- stimulus is greater than 0%. Consequently, in the extinction task, Irp2^−/−^ mice quickly learn that no reward is possible and inhibit their response to the stimulus. When a reward is possible, as in reversal learning, the Irp2^−/−^ mice struggle to reverse their responses to align with the new reward contingencies This difficulty in understanding changes in reward contingencies could result in a protracted interval during which Irp2^−/−^ mice are choosing the newly rewarded stimulus at or near the level expected due to chance.

## 5 Conclusion and future Directions

The results of this study establish motor, somatosensory, and behavioral deficits in Irp2^−/−^ mice, a model of iron metabolism misregulation thought to cause functional iron deficiency in neurons. The Irp2^−/−^ model significantly differs in severity from the IRP2 patients. Various reasons for the milder disease phenotype of the mouse model compared to human patients exist from differences in axon length, brain complexity, to reliance on mitochondrial function; but this is not uncommon for neurodegenerative diseases which often require gain-of-function or overexpression mutations to recapitulate the human phenotype in a mouse model. Nevertheless, the Irp2^−/−^ model offers the ability to examine this disease manifestations on a mechanistic level. The neurodegenerative disease in the IRP2 patients develops early with cerebral palsy, hypotonia, and seizures presenting in the first year. It's also likely that loss of IRP2 has immediate effects on neurodevelopment. The patients are also not discovered until they are referred to clinics that can perform genome sequencing to determine the cause of their neurological disease. These realities make the use of the patients as a model for iron metabolism misregulation difficult. The mildness of the Irp2^−/−^ mouse model however may represent an “early” snapshot of the disease. It is interesting that Irp2^−/−^ mice seem to have very specific cognitive defects. Learning and memory and anxiety appear to be unaffected by loss of Irp2, but Irp2^−/−^ mice were consistently hyperactive in novel environments and showed a pronounced reversal learning deficit. This specificity is unlikely to be true in the patients who fail to meet developmental milestones and are so impaired that it would be impossible to perform similar executive function tests, but these defects do offer a glimpse at which systems are most affected as well as offer insight into the effects of iron deficiency more generally. Our current understanding of this novel neurological disorder in humans is currently limited to a small number of patients presenting with the most severe outcomes of complete IRP2 ablation. The milder disease phenotype of the Irp2^−/−^ mice characterized in this study could be observed in human patients who have hypomorphic mutations of IREB2. However, combined with the frontal lobe atrophy of the IRP2 patients, however, the reversal learning impairment would indicate prefrontal cortex dysfunction in Irp2^−/−^ mice. The prefrontal cortex is highly dependent on monoamine systems creating circuits between the frontal cortex and subcortical structures. It's possible that these systems are particularly vulnerable to iron deficiency due to both tyrosine hydroxylase and tryptophan hydroxylase being iron-dependent proteins that are the rate-limiting steps of monoamine neurotransmitter synthesis. Thus, monoaminergic neurons may be particularly sensitive to the functional iron deficiency caused by ablation of Irp2 and may experience a “double hit” of mitochondrial dysfunction and disrupted neurotransmitter synthesis and/or signaling. Future work looking at disruptions in neurotransmitter signaling in Irp2^−/−^ mice could offer additional insights into the pathology observed in the IRP2 patients which could have potential therapeutic implications.

## Funding

This research was supported by the 10.13039/100030692Intramural Research Program (IRP) of the 10.13039/100000071National Institute of Child Health and Human Development (ZIA HD001602 to TAR), and in part by the 10.13039/100000025National Institute of Mental Health (ZIA MH002951 to YC) and Rodent Behavioral Core (ZIC MH002952 to YC).

## CRediT authorship contribution statement

**Christina Porras:** Conceptualization, Methodology, Validation, Formal analysis, Investigation, Writing – original draft, Visualization. **Hayden Olliviere:** Methodology, Investigation. **Sean P. Bradley:** Formal analysis, Visualization. **Alice M. Graham:** Methodology, Formal analysis. **Yogita Chudasama:** Funding acquisition, Resources, Writing – review & editing. **Tracey A. Rouault:** Conceptualization, Resources, Writing – review & editing, Supervision, Project administration, Funding acquisition, Resources.

## Declaration of competing interest

The authors declare that they have no known competing financial interests or personal relationships that could have appeared to influence the work reported in this paper.

## Data Availability

Data will be made available on request.
