# Supplementary material for: Ablation of Iron Regulatory Protein 2 produces a neurological disorder characterized by motor, somatosensory, and executive dysfunction in mice
Source: Curr Res Neurobiol. 2024 Aug 10;7:100136. doi: 10.1016/j.crneur.2024.100136 (PMC11372806; doi:10.1016/j.crneur.2024.100136)
Supplement: Multimedia component 2 [file mmc2.docx]

Supplementary Materials


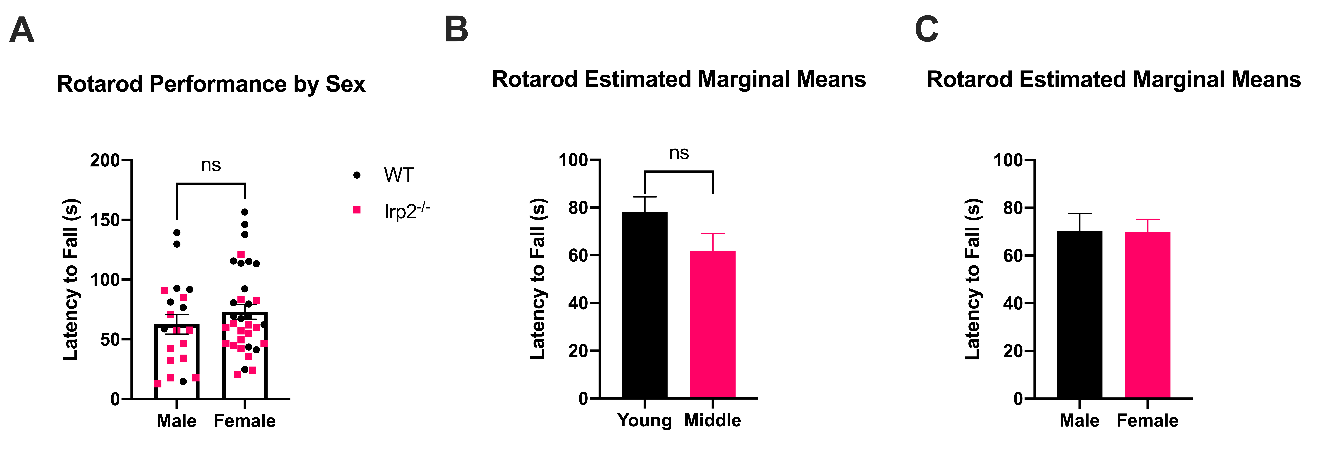


Figure S1.

A) An unpaired t-test did not detect a significant difference in rotarod performance based on sex. B-C) An ANCOVA analysis didn’t detect a significant difference in rotarod performance based on age or sex when controlling for weight (covariate evaluated at weight = 33.26).


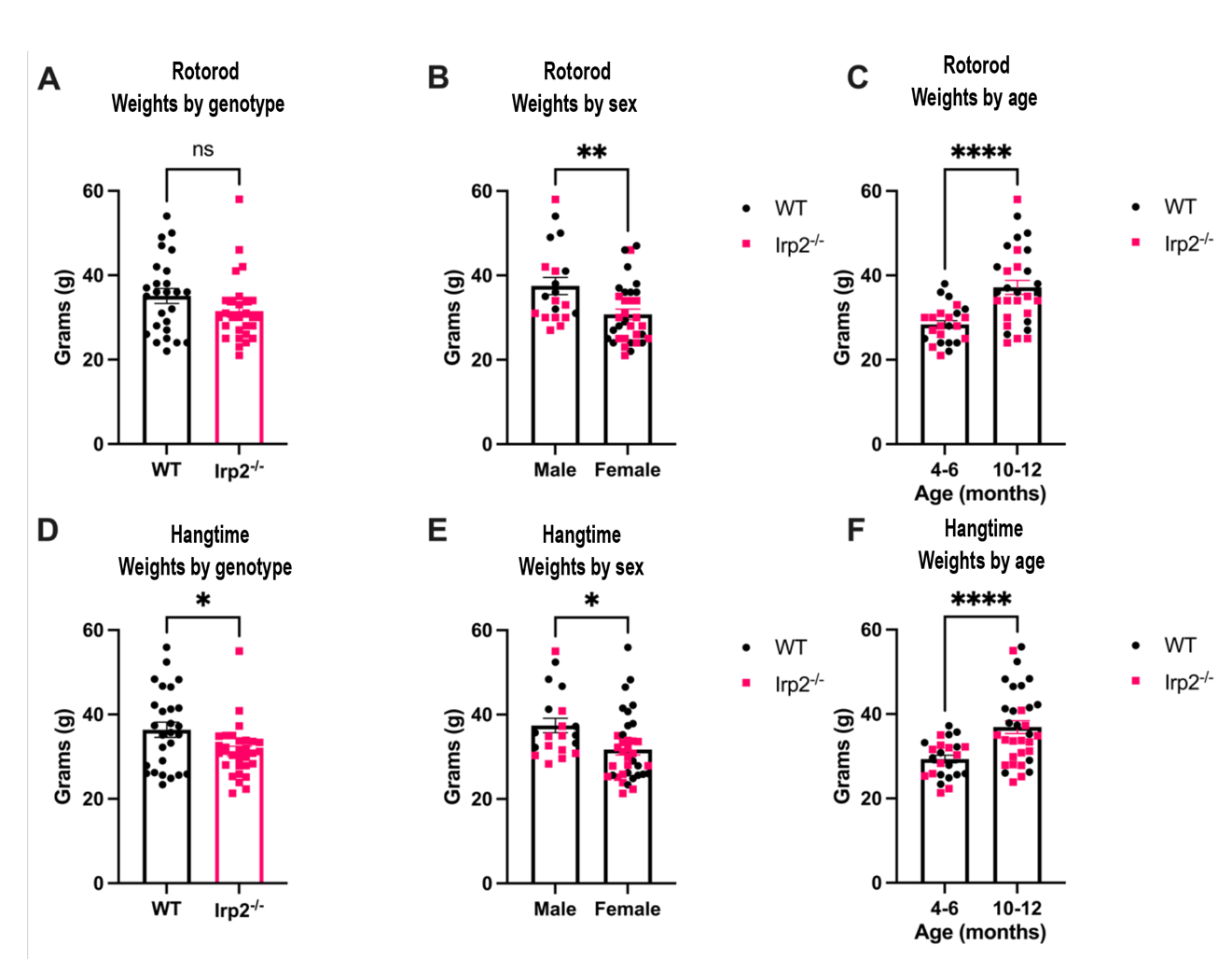


Figure S2: Weight Analysis for rotarod and hangtime grip test

A-C) Unpaired t-tests of the weights of WT and Irp2^-/-^ mice used for the rotarod showed a significant difference between male and female mice and young and middle-aged mice. D) Welch’s t-test of WT and Irp2^-/-^ mice used for the hangtime test revealed a significant decrease in the weight of Irp2^-/-^ mice. E) Unpaired t-test detected a significant decrease in the weight of female mice. F) Welch’s t-test of WT and Irp2^-/-^ showed a significant increase in middle-aged mice. * p<0.05; ** p<0.01; **** p<0.0001


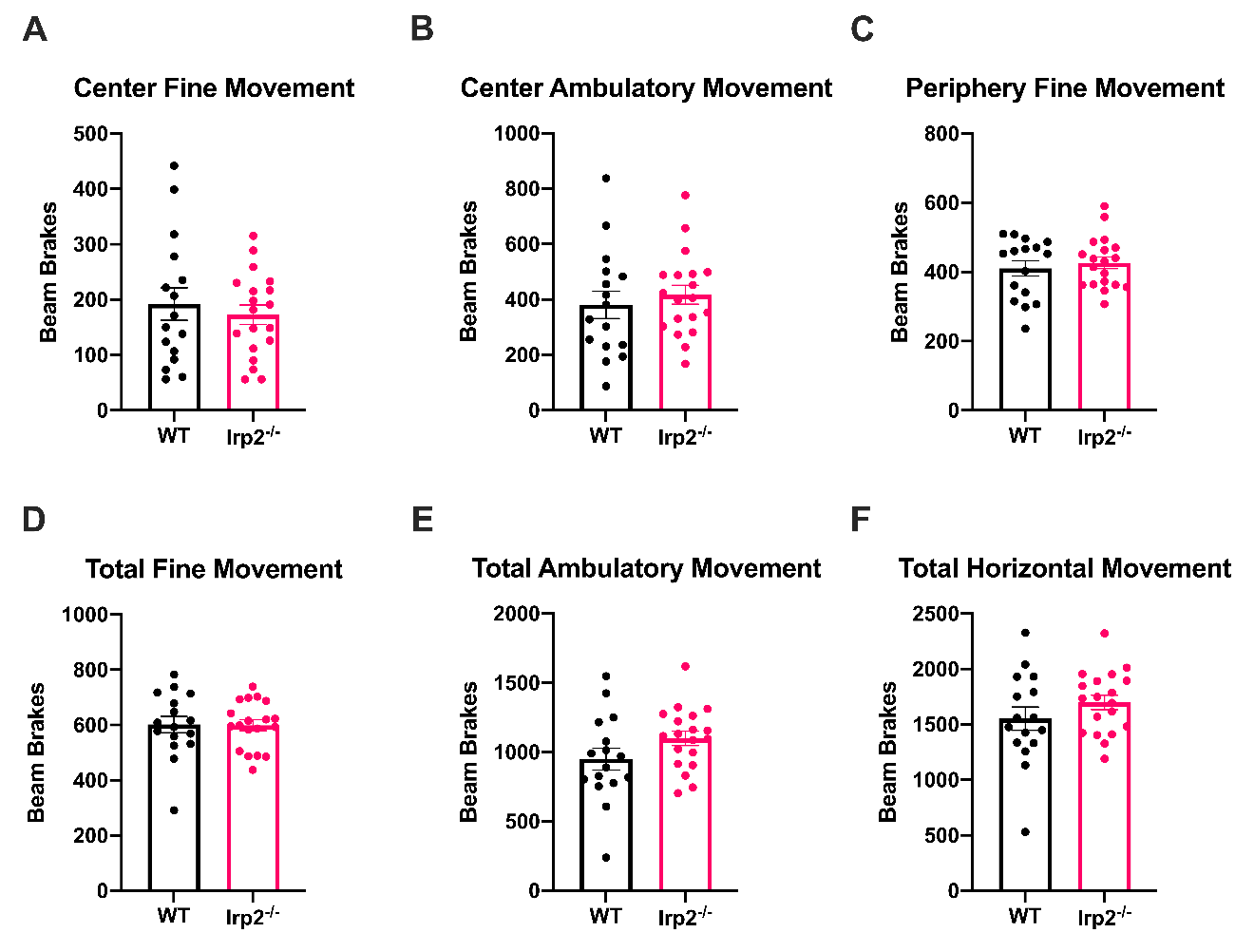


Figure S3: Additional open field metrics

A-D) Summary of Open Field metrics which didn’t differ significantly between WT and Irp2^–/–^. E-F) Total ambulatory and total horizontal movement had a slight increase due to the trend of increased periphery ambulatory movement included in these metrics.


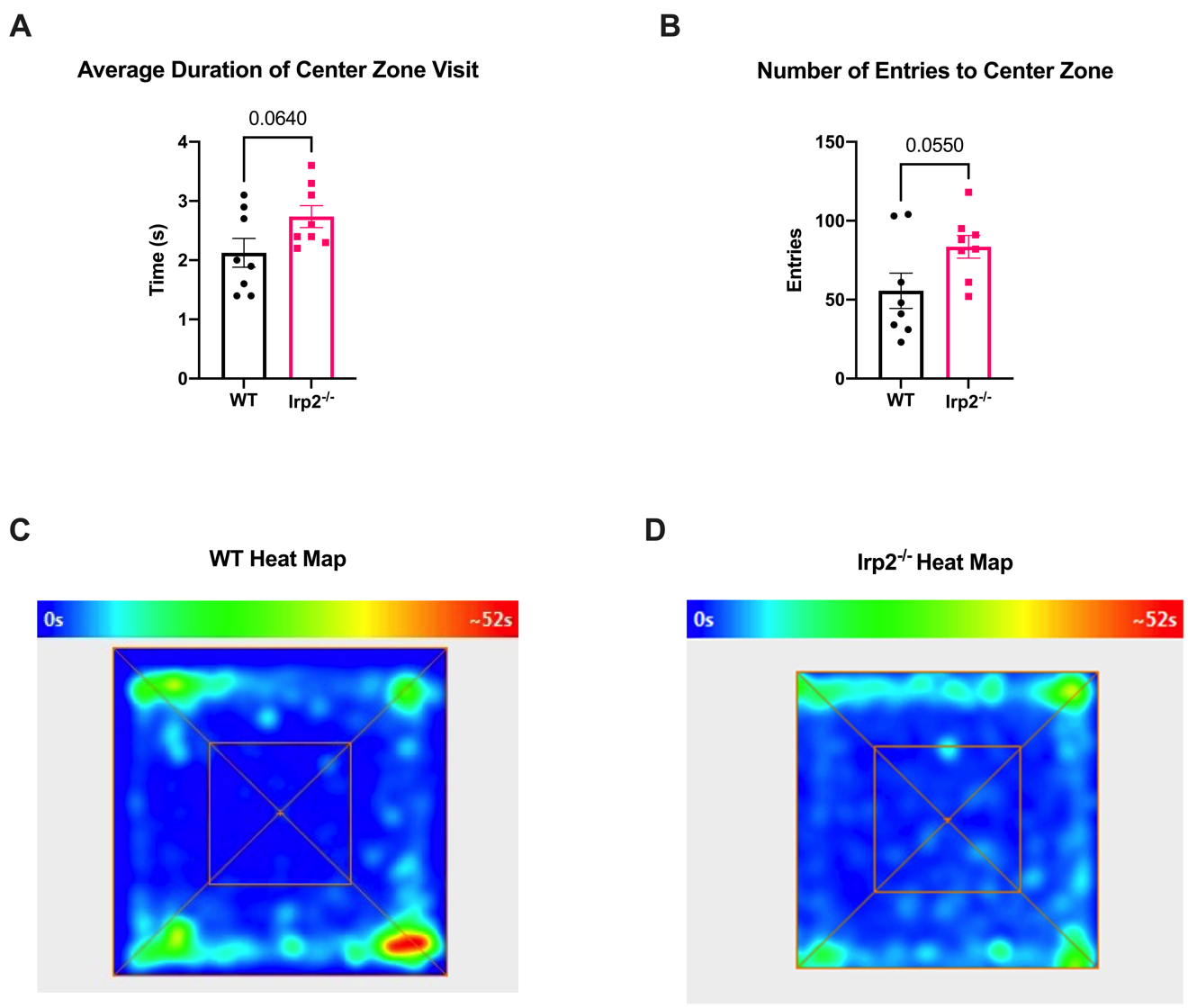


Figure S4: Open Field Anxiety Supplemental

A) Irp2^–/–^ mice tended to have a longer average duration of visit to the center zone. B) Irp2^–/–^ mice also tended to enter the center zone more often. C) WT Heat map of location during the trial. D) Irp2^–/–^ Heat map of location during the trial.


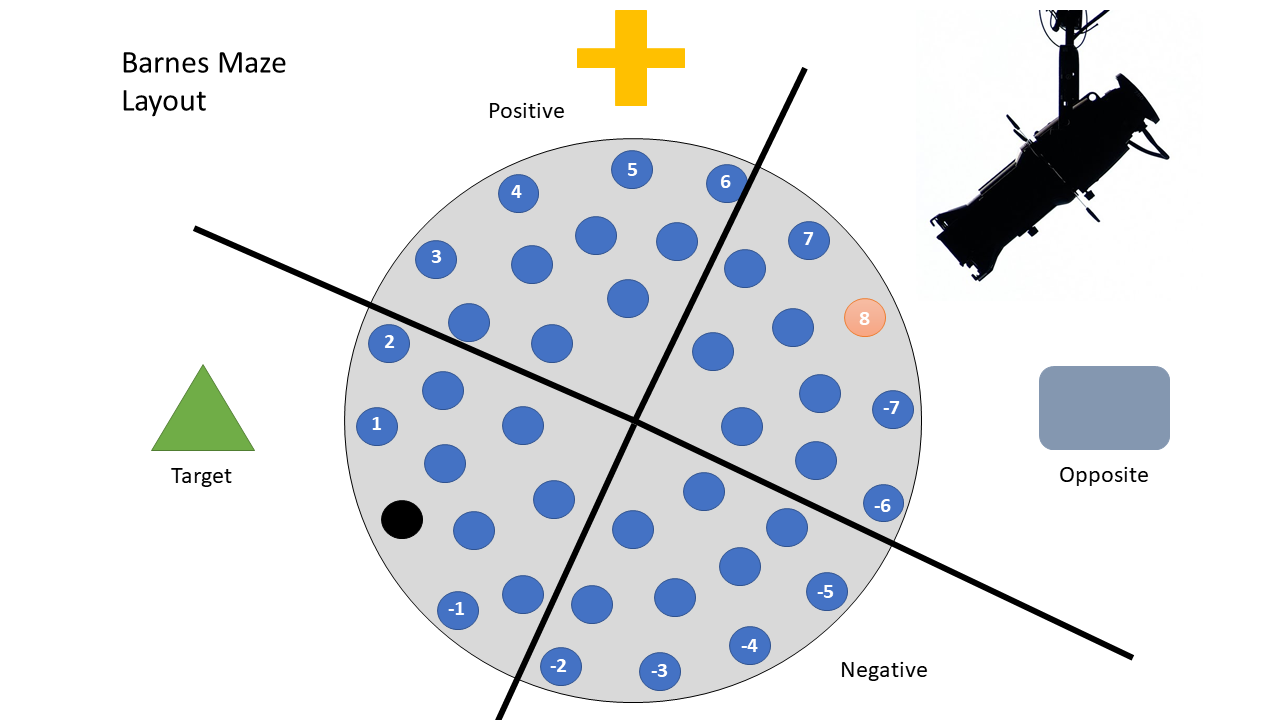


Figure S5: Barnes maze layout

An illustration of the experimental setup used for a Barnes maze test. A 40-hole elevated platform was placed in the center of the testing room. An overhead lamp was mounted above the platform to create an aversive environment. Three location cues (green triangle, yellow cross, blue square) were placed on the walls of the testing room. The maze was split digitally into four equal zones (target, positive, negative, and opposite) based on the location of the target hole (black circle). For reversal learning the location of the escape box was moved 180º (coral circle). Deviation scores (1-8) were based on outside hole’s distance from target hole.

Figure S6: Additional Barnes Maze Metrics

Unpaired (A, C-E, I-J) and Welch’s (B, F-H) t-tests were used to compare total distance (A-B), primary latency (C-D), primary distance (E-F), visits to target hole (G-H), and primary path efficiency (I-J) in acquisition and reversal probe trials. * p<0.05; ** p<0.01


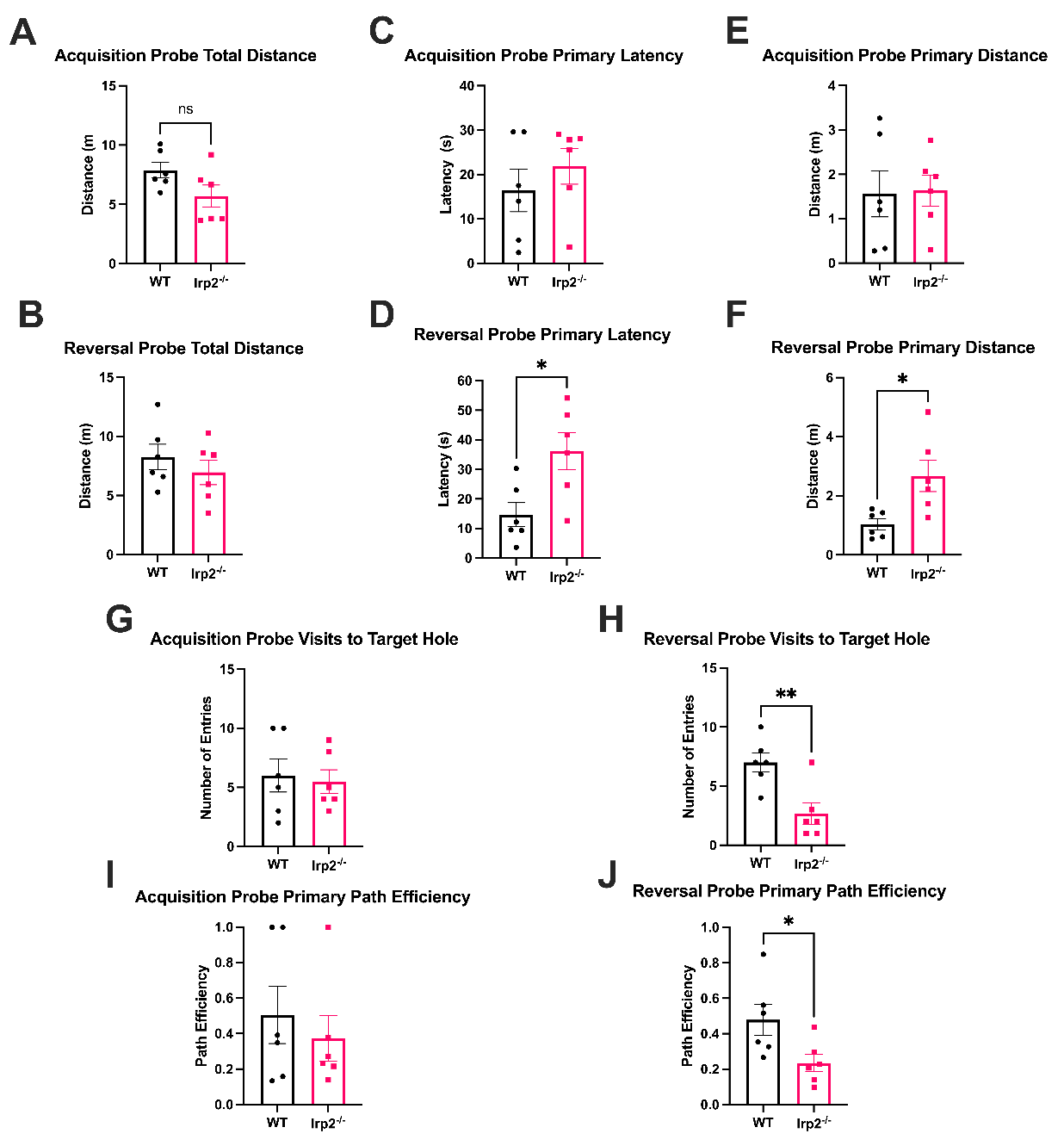


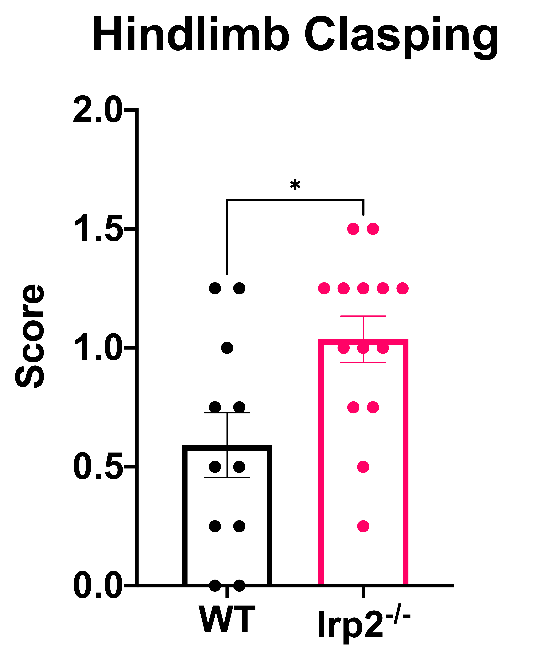


Figure S7: Hindlimb Clasping

Mice were lifted by the base of their tail for 10 seconds. If there was no hindlimb retraction to the abdomen, the mouse received a score of 0. If one hindlimb was retracted for more than 50% of the time, it received a score of 1. If both hindlimbs are partially retracted for more than 50% of the time, a score of 2; and if completely retracted, a score of 3. An unpaired t-test was used to compare WT and Irp2^–/–^ mice. * p<0.05

Figure S8: Progression of WT and Irp2^–/–^ mice through pretraining
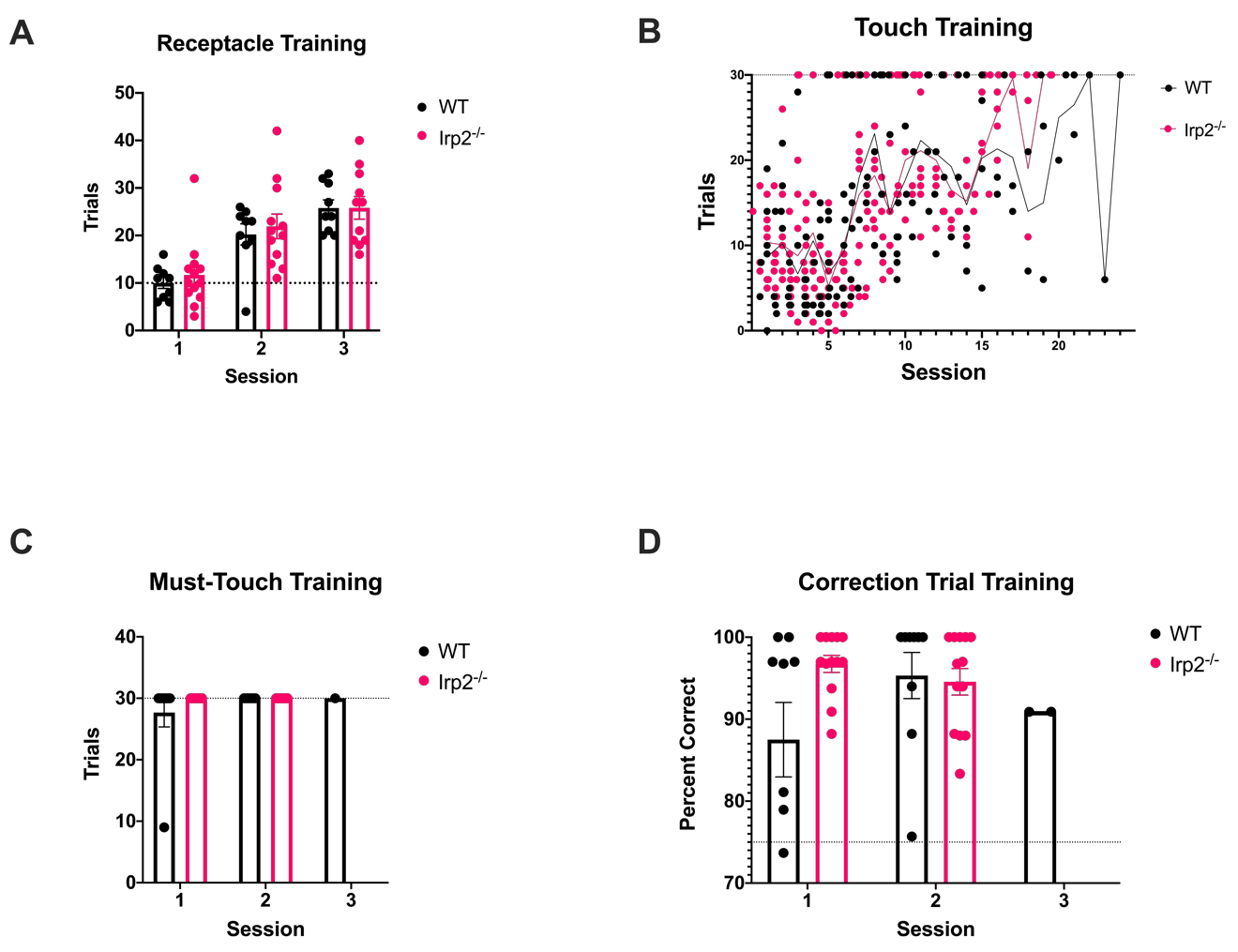


WT and Irp2^–/–^ mice progressed through A) receptacle training, B) touch training, C) must-touch training, and D) correction trial training at essentially the same pace. Dotted lines represent the criterion for each stage of training. Each time an individual mouse performed above criterion for two consecutive stages they progressed to the next stage.
