# Supplementary material for: Ablation of Iron Regulatory Protein 2 produces a neurological disorder characterized by motor, somatosensory, and executive dysfunction in mice
Source: Curr Res Neurobiol. 2024 Aug 10;7:100136. doi: 10.1016/j.crneur.2024.100136 (PMC11372806; doi:10.1016/j.crneur.2024.100136)
Supplement: Multimedia component 1 [file mmc1.pdf]

Peer Review Overview

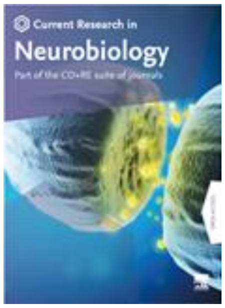

Manuscript Title: Ablation of Iron Regulatory Protein 2 produces a neurological disorder characterized by motor, somatosensory, and executive dysfunction in mice*

Received	May 06, 2023
1st Decision	Aug 14, 2023
1st Revision Submitted	Nov 07, 2023
2nd Decision	Dec 27, 2023
2nd Revision Submitted	Apr 30, 2024
3rd Decision	Jun 18, 2024
3rd Revision Submitted	Jul 15, 2024
Accepted	Jul 31, 2024

1st Decision letter

Reference: CRNEUR-D-23-00038
Title: Ablation of Iron Regulatory Protein 2 produces a neurological disorder characterized by motor, somatosensory, and executive dysfunction in mice*
Journal: Current Research in Neurobiology

Dear Dr Porras,

Two reviewers have completed their reviews and recommend reconsideration of your manuscript following revision. I invite you to resubmit your manuscript after addressing the comments below. Please resubmit your revised manuscript by Sep 28, 2023.

When revising your manuscript, please consider all issues mentioned in the reviewers' comments carefully: please outline in a cover letter every change made in response to their comments and provide suitable rebuttals for any comments not addressed. Please note that your revised submission may need to be re-reviewed.

Current Research in Neurobiology values your contribution and I look forward to receiving your revised manuscript.

Kind regards,

Christopher I. Petkov
Editor in Chief
Current Research in Neurobiology

Comments from Editors and Reviewers:

Reviewer 1:

This study investigates a subset of motor, sensory, cognitive and behavioral characteristics of a specific mouse model lacking iron regulatory protein 2 (Irp2-/-), which has been extensively backcrossed onto a C57Bl/6J background. They find significant impairments in motor function (rotarod and hang test) and somatosensory function (hot and cold plate), hyperactivity within maze environments (open field and Y-maze), as well as decrements in spatial learning/acquisition and reversal under aversive conditions (Barnes maze) and during the reversal learning phase of an appetitive touchscreen visual discrimination paradigm. While motor and sensory deficits have previously been reported in related Irp2-/- mouse models, the cognitive assessments are novel. The authors suggest that the Irp2-/- mouse model may represent an early, milder version of the human disease, with marked motor and sensory deficits alongside selective impairments in behavioral flexibility.

The behavioral testing in this manuscript appears be have been conducted carefully and rigorously. The procedures are well-established and described clearly and thoroughly. The selection of tests provides an both a broad and detailed analysis of the behavioral phenotype of the chosen Irp2-/- mouse model. Overall, the manuscript is well written and provides an interesting and original (for some domains) behavioral analysis of a specific Irp2-/- mouse model. However, given the observed general hyperactivity of Irp2-/- mice, I have several concerns regarding possible confounds in the motor and sensory assessment data, as well as in the analysis and interpretation of the Barnes maze and touch screen data.

Major points:

1) The rotarod is a test that involves multiple motor processes including motor learning, coordination and endurance. It would thus be useful to see the graph and analysis of performance across trials in order to help interpret these data. In addition, it is stated in the Methods that "Trials where the mouse jumped from the rotarod were excluded". Were these trials rerun to ensure each animal had 6 trials, or merely excluded outright? These excluded trials should be presented and analyzed as they may impact on interpretation of the results. This is particularly important given that the hyperactivity of Irp2-/- mice observed across the maze-based tests may confound motor assessment on the rotarod. Such a confound may help explain the somewhat paradoxical observation that the effect size for Irp2-/- rotarod impairment appears to be larger in younger-aged mice.

2) The grid hang test was performed on male and female WT and of Irp2-/- mice at two different age ranges. Why were the age-group data not presented and analyzed in the same way as for the rotarod, e.g., including age range as a predictive factor in Figure 2 and table G.9? Similar to the rotarod, the interpretation of motor differences in the grid hang test may be confounded if animals are impulsive/hyperactive and tend to jump from the grid. Was this behavior observed and recorded?

3) Hyperactivity and differences in gait, which have been previously reported in Irp2-/- mice, may confound interpretation of hind paw nociception in hot and cold plate tests. Was locomotor activity measured during the hot and cold plate tests in order to rule out this potential confound? Were any

other measures of nociception assessed that are less sensitive to such confounds?

4) As the data are currently presented, I am not convinced regarding the specificity of reversal learning deficits in either the Barnes maze or the touch screen visual discrimination and reversal tasks.

a) In the Barnes maze, the significant difference of largest effect size was the latency to find the target hole during initial task acquisition phase. Moreover, latency to find the target during reversal learning phase was no longer significant. These effects, across the bulk of learning trials, suggest a potential task early acquisition deficit that wanes over training rather than a selective deficit in reversal learning.

b) Also in the Barnes maze, the main evidence suggestive of a selective impairment is on the single probe trials after learning. However, the measure of time in the target zone during a probe can be an unreliable index of spatial learning performance, as it might reflect animals flexibly searching for a new target location after the escape box is not found in its expected location. This is particularly true for probes after reversal learning (e.g., after animals have learned that the escape location is not fixed). While a trend in first hole choice deviation score for the reversal probe trial is somewhat supportive, this measure can also be misleading without more detailed knowledge of path/search strategy. At minimum, the traditional measures of Barnes maze performance (including latency, distance, errors and proportion of mice finding the target location) should be presented, for both acquisition and probe trials, to help clarify and support the reported reversal probe results.

c) In the visual discrimination and reversal learning touchscreen tests, although the reversal learning phase was the only phase that revealed a significant difference between Irp2-/- and WT mice, there was a clear mean difference between groups during the acquisition phase. As reversal learning can be sensitive to the amount of acquisition training, the acquisition phase trials should be included as a factor or covariate in the analysis in order to ensure that the reversal learning effect is in fact selective (e.g., significant interaction of genotype x reversal phase when acquisition phase is included as a factor, or significant effect of genotype when acquisition phase is included as a covariate).

d) Also in the visual discrimination and reversal learning touchscreen tests, additional performance measures should be provided to ensure that there are no ancillary factors that might be contributing to the observed results. These include response and collection latencies, as well as the number of correction trials and the total trials per session (e.g., if total trials are spread across a greater number of sessions for one genotype, this might bias the interpretation of reversal learning results).

Minor points:
5) As body weight is used as a covariate, and was a statistically significant regressor in the motor analyses, it is important to include graphs and analyses of the raw body weights taken in parallel with the motor testing.
6) What were the total activity and number of entries into the open/closed sections of the elevated zero maze? These will help better interpret performance on this test.
7) Figures 4B and 8A in which significance stars are positioned over individual data points suggesting a significant interaction, however, no significant interaction effect was observed in the ANOVA. These should be removed and the appropriate main effects indicated instead.

8) Was sphericity measured and accounted for in the repeated measures ANOVA data presented in the manuscript.

Reviewer 2:

The authors characterized the Irp2 ablation mice in different genetic background. It is worth further clarifying the importance of IRP2 in the CNS system since IRP2 gene mutation causes a complex neurological disorder. The methods cover the various aspects concerning CNS functions. It is great. However, the manuscript can be improved. The major concerns are:
1. The manuscript is too long, and feel very redundant. Make it short and concise.
2. There is a lot of discussion in Results. Please remove it.
3. The style is oral and casual English. Make it somehow more formal.
4. For significance, $p<0.05$ is considered significant. Therefore, many sentences must be softened, removing the "strong" and other too-strong words.
5. Mitochondrial dysfunction is supposed to be a big problem in Irp2 ablation mice and patients. This mechanism can be discussed more.
The minors are:
1. In Results: mistakenly paragraphed twice.
2. Many "have/had" can be replaced by "show/showed". This might be casual-English problems.
3. The manuscript is easy to follow but has many language errors.

1st Author Response Letter

Response to comments from Editors and Reviewers:

Reviewer 1
Major points: 1) The rotarod is a test that involves multiple motor processes including motor learning, coordination and endurance. It would thus be useful to see the graph and analysis of performance across trials in order to help interpret these data. In addition, it is stated in the Methods that "Trials where the mouse jumped from the rotarod were excluded". Were these trials rerun to ensure each animal had 6 trials, or merely excluded outright? These excluded trials should be presented and analyzed as they may impact on interpretation of the results. This is particularly important given that the hyperactivity of Irp2-/- mice observed across the maze-based tests may confound motor assessment on the rotarod. Such a confound may help explain the somewhat paradoxical observation that the effect size for Irp2-/- rotarod impairment appears to be larger in younger-aged mice.

Response: A graph and analysis of performance across trials has been added to the supplemental figure for the rotarod test. Only one mouse had a trial excluded due to jumping. Because we observed an increase in rotarod performance over subsequent trials ($p<0.0001$), we felt excluding the 5th trial of this mouse and averaging the values of trials 1-4 and 6 was more accurate than having the mouse complete a 7th trial. If necessary, we could exclude this mouse completely from the analysis.

2) The grid hang test was performed on male and female WT and of Irp2-/- mice at two different age ranges. Why were the age-group data not presented and analyzed in the same way as for the rotarod, e.g., including age range as a predictive factor in Figure 2 and table G.9? Similar to the rotarod, the

interpretation of motor differences in the grid hang test may be confounded if animals are impulsive/hyperactive and tend to jump from the grid. Was this behavior observed and recorded?

*Response: Age was not used as a predictive factor because of unequal variances across age groups which was not seen in the rotarod test likely due to the 60s cutoff on the hangtime test. The ANCOVA test was used to demonstrate that differences in raw hangtime/rotarod performance could indicate a genotype*sex effect or genotype*age effect in Irp2-/- mice, but when weight was used as a covariate only a main genotype effect is significant. This is important because the Irp2-/- phenotype was previously described as progressive, so other groups tested older and likely overweight male mice. Our argument is that in the future only young (6 months or less) male and female mice should be used in motor tests of Irp2-/- models.*

The grids were lightly shaken to encourage the mice to grip the wire cage. It is impossible when the mice fall from the grid to accurately determine if it was due to the mouse losing their grip of the wire or because they chose to let go/jump. Hyperactivity/impulsivity could increase the latter. This was why our group performed both the hangtime and rotarod tests to look at the motor phenotype of Irp2-/- mice. The hangtime test allowed us to compare our results with previous work done by our group with a different Irp2-/- model, and the rotarod test is less sensitive to hyperactivity/impulsivity confounds because it is easier to distinguish between jumping and falling. It is also the more traditional test for motor function/coordination. Together both tests demonstrate a very significant motor defect.

3) Hyperactivity and differences in gait, which have been previously reported in Irp2-/- mice, may confound interpretation of hind paw nociception in hot and cold plate tests. Was locomotor activity measured during the hot and cold plate tests in order to rule out this potential confound? Were any other measures of nociception assessed that are less sensitive to such confounds?

Response: The setup for the cold and hot plate analysis did not allow for accurate measurements of locomotor activity. We recorded the trials from the side instead of from the top, so tracking of distance would be impossible or very inaccurate. The purpose of these tests was to compare our model's performance with another group's previously published difference in hot plate nociception, so we chose a similar procedure and setup. Hyperactivity could increase the number of nociceptive behaviors and decrease latency, but we observed the opposite trend in both hot and cold plate tests. Differences in gait/motor defects could decrease behaviors and increase latency especially jumping in the cold plate test, but any attempt was marked as a jump, and the motor phenotype would be less likely to affect number/latency of licks in the hot plate test.

We are currently working to establish a collaboration with a group that specializes in somatosensory function to conduct more accurate measures of nociception and to do a comprehensive study of periphery sensory neurons that is similar to what our group did previously with lower motor neurons in Irp2-/- mice. The reviewer's concerns have been added to the manuscript in the discussion of the hot/cold plate results.

4) As the data are currently presented, I am not convinced regarding the specificity of reversal learning deficits in either the Barnes maze or the touch screen visual discrimination and reversal tasks.

a) In the Barnes maze, the significant difference of largest effect size was the latency to find the target hole during initial task acquisition phase. Moreover, latency to find the target during reversal learning

phase was no longer significant. These effects, across the bulk of learning trials, suggest a potential task early acquisition deficit that wanes over training rather than a selective deficit in reversal learning.

b) Also in the Barnes maze, the main evidence suggestive of a selective impairment is on the single probe trials after learning. However, the measure of time in the target zone during a probe can be an unreliable index of spatial learning performance, as it might reflect animals flexibly searching for a new target location after the escape box is not found in its expected location. This is particularly true for probes after reversal learning (e.g., after animals have learned that the escape location is not fixed). While a trend in first hole choice deviation score for the reversal probe trial is somewhat supportive, this measure can also be misleading without more detailed knowledge of path/search strategy. At minimum, the traditional measures of Barnes maze performance (including latency, distance, errors and proportion of mice finding the target location) should be presented, for both acquisition and probe trials, to help clarify and support the reported reversal probe results.

Response (a and b): The requested additional metrics (primary latency and primary and total distance) have been added to a supplemental figure. Primary path efficiency i.e., the primary distance to the target hole divided by the shortest possible distance was used instead of search strategy/errors because it is a quantitative measurement that incorporates both. For example, mice with random search strategies or a high number of errors would have a lower path efficiency (closer to 0) compared to mice with a direct search strategy with fewer errors (closer to 1). We also included the number of visits to the target hole. All mice found the target hole location, so a proportion metric was unnecessary. These measurements are given for both acquisition and reversal probe trials. These are also discussed in the text of the manuscript. Consistent with what was previously provided, in these additional measures of performance a small but insignificant effect size of genotype in acquisition trials may be indicative of an early task acquisition deficit that wanes over time, but the effect size increases during reversal learning even though the Irp2-/- mice should be more familiar with the task and the environment is no longer novel (reducing possible effects of their hyperactive phenotype). If only an early acquisition deficit was affecting performance the effect size should stay the same or decrease over subsequent training/probe trials. This suggests that though it may take Irp2–/– mice longer to learn the initial target hole location (though this is only indicated by insignificant increases in primary latency which could be influenced by hyperactivity/impulsivity in Irp2–/– mice and not actually the result of a learning deficit), once a second solution is presented Irp2–/– mice fail to adjust their behavior e.g. inhibit checking of initial target hole (decrease in mean deviation score) or focus on checking the new target hole (decrease in primary path efficiency/fewer number of visits) even though they are able to learn its location (decrease in primary latency in reversal training over the three days/100% of mice visiting the target hole in the probe trial).

c) In the visual discrimination and reversal learning touchscreen tests, although the reversal learning phase was the only phase that revealed a significant difference between Irp2-/- and WT mice, there was a clear mean difference between groups during the acquisition phase. As reversal learning can be sensitive to the amount of acquisition training, the acquisition phase trials should be included as a factor or covariate in the analysis in order to ensure that the reversal learning effect is in fact selective (e.g., significant interaction of genotype x reversal phase when acquisition phase is included as a factor, or significant effect of genotype when acquisition phase is included as a covariate).

Response: The suggested analysis would be difficult to do due to unequal variances between groups. Also, sessions to criterion is not the best metric for comparison of touchscreen performance. Therefore, to address the reviewer's concerns, we did a comparison of WT and Irp2-/- performance during the first three sessions of both acquisition and reversal learning stages. All mice completed at least three sessions

of acquisition training before some mice advanced to reversal learning due to meeting the criterion. We did a three-way ANOVA analysis using session, genotype, and stage (acquisition or reversal) as factors. As expected, there was a very significant ($p < 0.0001$) session effect. Stage and genotype effects were also significant ($p < 0.01$). The session x stage interaction effect was very significant ($p < 0.0001$). Session x genotype effect was not significant ($p=0.28$). Stage x genotype effect was nearly significant ($p=0.09$), and the session x stage x genotype interaction effect was not significant ($p=0.74$). The stage x genotype effect being nearly significant would indicate that the genotypes performed differently depending on the stage of training, and that the difference in reversal learning performance is due to a reversal learning deficit not solely a general acquisition deficit. Similarly, to the Barnes maze performance, our interpretation of the results is that a small effect of genotype may cause a learning delay in the initial acquisition stages, but this effect increases in the reversal stage because Irp2-/- mice struggle to inhibit responding to a previously rewarded stimulus and choose a previously unrewarded stimulus as seen in an increase both in perseverative and new learning errors. As can be seen in the added graph to Figure 10, the highest performing mice by Session 3 are WT mice in the reversal stage (likely due to familiarity with the task). The lowest performing mice are Irp2-/- mice also during the reversal stage. Essentially the effect of genotype increases during subsequent reversal training instead of decreasing or staying the same which would be expected with an early acquisitional deficit/general learning disability.

d) Also in the visual discrimination and reversal learning touchscreen tests, additional performance measures should be provided to ensure that there are no ancillary factors that might be contributing to the observed results. These include response and collection latencies, as well as the number of correction trials and the total trials per session (e.g., if total trials are spread across a greater number of sessions for one genotype, this might bias the interpretation of reversal learning results).

Response: Our colleague that helped with the touchscreen tests is preparing a report of these measures. It was not able to be completed by the Revision due date, but we can add them at a later edit and include them in a supplemental figure.

Minor points:
5) As body weight is used as a covariate, and was a statistically significant regressor in the motor analyses, it is important to include graphs and analyses of the raw body weights taken in parallel with the motor testing.

Response: Graphs and analysis of body weights for the rotarod and hangtime testing has been added to a new supplemental figure.

6) What were the total activity and number of entries into the open/closed sections of the elevated zero maze? These will help better interpret performance on this test.

Response: Total distance and distance travelled in the open/closed sections as well as number of entries into open/closed sections indicated that Irp2-/- mice were hyperactive in the elevated zero maze, but the percentage of time spent in the open sections was not different between Irp2-/- and WT mice indicating no difference in anxiety/avoidance of open sections. These metrics have been added to the elevated zero maze figure.

7) Figures 4B and 8A in which significance stars are positioned over individual data points suggesting a significant interaction, however, no significant interaction effect was observed in the ANOVA. These should be removed, and the appropriate main effects indicated instead.

Response: significance stars have been removed and main effects indicated in text.

8) Was sphericity measured and accounted for in the repeated measures ANOVA data presented in the manuscript.

Response: Sphericity was not assumed, and Geisser-Greenhouse corrections were used.

Reviewer 2

The authors characterized the Irp2 ablation mice in different genetic background. It is worth further clarifying the importance of IRP2 in the CNS system since IRP2 gene mutation causes a complex neurological disorder. The methods cover the various aspects concerning CNS functions. It is great. However, the manuscript can be improved. The major concerns are:
1. The manuscript is too long, and feel very redundant. Make it short and concise.
2. There is a lot of discussion in Results. Please remove it.
3. The style is oral and casual English. Make it somehow more formal.
4. For significance, $p<0.05$ is considered significant. Therefore, many sentences must be softened, removing the "strong" and other too-strong words.

Response (1-4/minors): Reviewer 2 listed several style concerns. We have incorporated their feedback and made additional changes to the manuscript. However, Reviewer 1 did not have any concerns on the length of the manuscript and requested additional information be added. The extra discussion in the results section is to explain the choice of behavioral tests. Behavioral characterization of Irp2-/- mice has been limited to motor testing, open field, and hot plate tests by groups that do not focus on behavioral research, so additional explanation of the other tests is necessary for other researchers in the field of iron metabolism/IRP proteins.

5. Mitochondrial dysfunction is supposed to be a big problem in Irp2 ablation mice and patients. This mechanism can be discussed more.

Response: The association with Irp2 ablation and mitochondrial dysfunction is mentioned multiple times in the manuscript (highlighted in yellow) with references to previous studies/reviews on the topic. We do not think it was appropriate to discuss this more because this study does not include mitochondrial function assays/tests.

The minors are:
1. In Results: mistakenly paragraphed twice.
2. Many "have/had" can be replaced by "show/showed". This might be casual-English problems.
3. The manuscript is easy to follow but has many language errors.
See first response to reviewer.

2nd Decision letter

Reference: CRNEUR-D-23-00038
Title: Ablation of Iron Regulatory Protein 2 produces a neurological disorder characterized by motor, somatosensory, and executive dysfunction in mice*
Journal: Current Research in Neurobiology

Dear Dr Porras,

The reviewers have reconsidered the revised manuscript and based on their input the revision effort is not sufficient to warrant consideration further. One of the reviewers has taken the time to substantiate why the conclusions do not seem to relate well to the data or analyses provided.

Although we would typically not consider a manuscript further if the first revision effort does not go much of the way towards addressing the reviewer concerns, in this case if the authors feel that they can address the remaining concerns of the reviewer below, we can consider a substantially revised, clarified and strengthened paper.

Therefore, I can invite you to resubmit your manuscript after addressing the comments below. Please resubmit your revised manuscript when it is sufficiently ready to be reconsidered.

Current Research in Neurobiology values your contribution and I look forward to receiving your revised manuscript.

Kind regards,

Christopher I. Petkov
Editor in Chief
Current Research in Neurobiology

Comments from Editors and Reviewers:

Reviewer 1:

The authors make numerous changes to improve their manuscript but have omitted many of the requested changes that are essential for proper interpretation of the behavioral data. I thus still have major concerns that the title and main conclusions are not supported by the data as they are currently presented.

1) As requested in the first review, it would be useful to see the graph(s) of rotarod performance across trials (with trial number as x-axis) to view the learning performance curves of each of the subgroups [e.g., genotype, sex, age]. These graph(s) and analysis do not seem to be included in the supplemental figures as stated.

2) As requested in the first review, the statistical analysis of the hang test, including all of the preselected independent variables [genotype, sex, age, weight as covariate], should be reported according to the original study design and hypotheses. An increase in variability across age groups should not preclude analysis by age on the grid hang test. Indeed, the results across age should be considered when interpreting the results (e.g., if young adult mice tend to have lower hang times than older mice this may indicate that that the test may be influenced by non-strength-related behaviors such as hyperactivity or jumping). Moreover, the hang test data are extremely limited and non-normally distributed due to severe ceiling effects caused by the 60-s time limit. They should be analyzed using the appropriate transformation, error and link function distributions and/or nonparametric techniques to

ensure that statistical assumptions are met. Perhaps it would be preferable to first increase the test time limit to avoid clear ceiling effects and/or to use other behavioral measures to validate the findings (e.g., using a quantitative grip strength apparatus) before recommending to restrict testing of older Irp2-/- mice on the hang test.

3) Hyperactivity or excessive movement on the rotarod and hang time tests might reduce performance independently of deficits in motor learning or strength. Similarly, hyperactivity might decrease observation of nociceptive behaviors due to less time for paws spent in continuous contact with the hot or cold surfaces or otherwise engaging in other movements. Can the possible confounding influence of hyperactivity be ruled out for these motor or sensory tests? If not, the results and conclusions from all of these tests should be described more tentatively.

4) An interpretation of a selective reversal learning deficit in the Barnes maze is not supported by the data as currently presented.
I do not see any of the additional requested measures (e.g., distance, errors) for the acquisition and reversal learning trials of the Barnes maze (only for the probe trials). These are essential for proper interpretation of the results. It is also important to include errors as well as distance for both acquisition and probe trials - errors do not always correlate with path distance and can help inform on search strategy.
It is unclear what the path efficiency metric is adding as all trials have the same central starting point. Addition of more common search strategy metrics (e.g., random, serial along the inner or outer rings, correction/landmark-based, or direct) would provide more useful description of any systematic performance differences between groups.
The interpretation of the results as a selective reversal learning deficit still appears to rely entirely on the single probe trials, while ignoring the training trials which comprise the bulk of the Barnes maze testing. The training trials during the acquisition and reversal stages are the critical to the interpretation of Barnes maze performance. Irp2-/- mice took significantly longer latencies to find the target escape hole across the 12 acquisition training trials than wt mice (the data for distance and errors during acquisition has still not been provided). No significant difference in latency between groups was observed during reversal learning training trials (distance and errors still not provided), with the largest mean difference between genotypes on the first day. This pattern of results clearly indicates that there is an initial learning impairment in Irp2-/- mice, but no statistical evidence of a deficit in reversal learning. This is the opposite of what is concluded.
It is important to note that the Barnes maze probe tests (with no escape box) are implemented primarily to examine whether mice are reliant on local cues from the escape box to help guide their behavior. Thus, an impairment only on the reversal probe test, but not during training, suggests that, relative to wt mice, Irp2-/- mice may have more learned more preferentially to depend on the escape box to locate the target hole. Critically, the reversal probe trial results on their own do not imply a specific deficit in reversal learning or cognitive flexibility, particularly when there are no significant deficits observed during reversal learning training.

5) An interpretation of a selective reversal learning deficit in the touchscreen tests is also not supported by the data as currently presented.
Unequal variances between groups does not preclude performing analysis of reversal learning while

accounting for possible differences in visual discrimination learning (by ANOVA or GLM regression). This analysis should be performed on the data for sessions to criterion, as well as on errors and trials to criterion (with and without correction trials). The authors should also include the key response latency measures as previously requested. In the absence of these analyses, it is impossible to interpret the results as suggesting a selective reversal learning deficit.
Moreover, even with the new truncated analysis performed by the authors - including only the first 3 visual discrimination and reversal learning sessions - a significant main effect of genotype was found with NO significant stage (discrimination vs reversal) X genotype interaction. As it stands, there is no evidence of a selective reversal learning deficit in Irp2-/- mice that is independent of a more general learning deficit.

6) Given that the statistical evidence appears to suggest a more general deficit in the Barnes maze and touchscreen tests, it is important to rule out possible nonspecific motor or sensory differences that might confound task performance. As described above, hyperactivity might contribute to many of the observed deficits in Irp2-/- mice across this study. In addition, a visual impairment in Irp2-/- mice might also explain the lower touchscreen performance (main effect) as well as deficits in learning and reliance on local escape box cues in the Barnes maze. Given the well-established role of iron on retinal function and other aspects of visual processing, have the basic visual capabilities of these Irp2-/- mice been assessed and ruled out?

2nd Author Response Letter

Response to comments from Editors and Reviewers:

Reviewer 1

Major points:
1) The rotarod is a test that involves multiple motor processes including motor learning, coordination and endurance. It would thus be useful to see the graph and analysis of performance across trials in order to help interpret these data. In addition, it is stated in the Methods that "Trials where the mouse jumped from the rotarod were excluded". Were these trials rerun to ensure each animal had 6 trials, or merely excluded outright? These excluded trials should be presented and analyzed as they may impact on interpretation of the results. This is particularly important given that the hyperactivity of Irp2-/- mice observed across the maze-based tests may confound motor assessment on the rotarod. Such a confound may help explain the somewhat paradoxical observation that the effect size for Irp2-/- rotarod impairment appears to be larger in younger-aged mice.

Response: We agree that an analysis of performance across trials be a useful addition to the dataset. In motor tasks like rotarods, there is a strong learning effect with time. Of the tested animals, only one mouse had a trial excluded due to jumping. Because we observed an increase in rotarod performance over subsequent trials ($p<0.0001$) , we felt excluding the 5th trial of this mouse and averaging the values of trials 1-4 and 6 was more accurate than having the mouse complete a 7th trial. For this reason, we did not analyze trials across learning, but we have clarified in the text that only one animal jumped off the rotarod (page 7). In addition, in the supplementary materials, we have added graphs to highlight the lack of difference as a function of sex and age when controlling for weight (Figure S1). We should also point

out that during the revision of this paper, the hyperactive account of the Irp2 null mice became less convincing so we have removed reference to this in the manuscript.

2) The grid hang test was performed on male and female WT and of Irp2-/- mice at two different age ranges. Why were the age-group data not presented and analyzed in the same way as for the rotarod, e.g., including age range as a predictive factor in Figure 2 and table G.9? Similar to the rotarod, the interpretation of motor differences in the grid hang test may be confounded if animals are impulsive/hyperactive and tend to jump from the grid. Was this behavior observed and recorded?

Response: Age was not used as a predictive factor because of unequal variances across age groups. This variance was not observed in the data for the rotarod test. We think this is likely due to the 60s cutoff time for the hangtime. We applied the ANCOVA model, as with the rotarod analysis, with weight as the covariate. This demonstrated a difference both in weight ($F_{weight}(1,52) = 14.438$, $p < 0.05$; Figure S2) and group ($F_{genotype}(1,52) = 14.438$, $p < 0.05$; Figure 2) in their latency to fall during the hangtime test. This finding is important because the Irp2-/- phenotype was previously described as progressive, so other groups tested older and likely overweight male mice. Our argument is that future projects should focus on young (6 months or less) male and female mice when assessing motor deficits in Irp2-/- models. We have now mentioned this in the text (page 20).
Note also that the grids were lightly shaken to encourage the mice to grip the wire cage. It is difficult to determine with precision whether mice lose their grip of the wire or because they choose to let go/jump. Assessing the animals on the grid hangtime and rotarod tests allowed us to compare our results with previous work done by our group with a different Irp2-/- models. In addition, in the rotarod test is less it is easier to distinguish between jumping and falling. Together, both tests demonstrate a motor deficit in the Irp2 ablated mice.

3) Hyperactivity and differences in gait, which have been previously reported in Irp2-/- mice, may confound interpretation of hind paw nociception in hot and cold plate tests. Was locomotor activity measured during the hot and cold plate tests in order to rule out this potential confound? Were any other measures of nociception assessed that are less sensitive to such confounds?

Response: The setup for the cold and hot plate analysis did not allow for accurate measurements of locomotor activity. We recorded the trials from the side rather than above to enable accurate tracking. The purpose of these tests was to compare our model's performance with another group's previously published difference in hot plate nociception, so we chose a similar procedure and setup. We agree that hyperactivity could increase the number of nociceptive behaviors and decrease latency, but we observed the opposite trend in both hot and cold plate tests. Differences in gait/motor defects could decrease behaviors and increase latency especially jumping in the cold plate test, but any attempt was marked as a jump, and the motor phenotype would be less likely to affect number/latency of licks in the hot plate test. We have now commented on this in the discussion (page 40).

4) As the data are currently presented, I am not convinced regarding the specificity of reversal learning deficits in either the Barnes maze or the touch screen visual discrimination and reversal tasks.

a) In the Barnes maze, the significant difference of largest effect size was the latency to find the target hole during initial task acquisition phase. Moreover, latency to find the target during reversal learning phase was no longer significant. These effects, across the bulk of learning trials, suggest a potential task early acquisition deficit that wanes over training rather than a selective deficit in reversal learning.

b) Also in the Barnes maze, the main evidence suggestive of a selective impairment is on the single probe trials after learning. However, the measure of time in the target zone during a probe can be an unreliable index of spatial learning performance, as it might reflect animals flexibly searching for a new target location after the escape box is not found in its expected location. This is particularly true for probes after reversal learning (e.g., after animals have learned that the escape location is not fixed). While a trend in first hole choice deviation score for the reversal probe trial is somewhat supportive, this measure can also be misleading without more detailed knowledge of path/search strategy. At minimum, the traditional measures of Barnes maze performance (including latency, distance, errors and proportion of mice finding the target location) should be presented, for both acquisition and probe trials, to help clarify and support the reported reversal probe results.

Response (a and b): As requested, we have included additional metrics in the main figure for the Barnes maze (figure 8) and included additional data in a supplemental figure (figure S6). We have also re-written the results section for the Barnes maze (section 3.7) and can confirm that there is no learning deficit during the acquisition phase (Fig 8A). Our data show that during acquisition, the Irp2 ablated mice were not impaired in finding the escape hole but were slow in finding the escape hole and made few errors in non-escape holes during the probe (Figure 8B).

When the escape hole was moved to the opposite location (reversal stage), the main difficulty with the Irp2–/– mice was their failure to visit the new location, and they spent less time in the target zone searching for the absent escape hole during the probe. Of particular interest was the high error rate in holes closer to the initial target hole combined with fewer visits and time spent in the new target zone. These data indicate that the Irp2 null animals were impaired in their search strategy causing a difficulty for that animal to adapt to environmental change. We have now corrected our results section to accommodate these changes (Pages 30-33) and discussion (pages 41-42).

c) In the visual discrimination and reversal learning touchscreen tests, although the reversal learning phase was the only phase that revealed a significant difference between Irp2-/- and WT mice, there was a clear mean difference between groups during the acquisition phase. As reversal learning can be sensitive to the amount of acquisition training, the acquisition phase trials should be included as a factor or covariate in the analysis in order to ensure that the reversal learning effect is in fact selective (e.g., significant interaction of genotype x reversal phase when acquisition phase is included as a factor, or significant effect of genotype when acquisition phase is included as a covariate).

d) Also in the visual discrimination and reversal learning touchscreen tests, additional performance measures should be provided to ensure that there are no ancillary factors that might be contributing to the observed results. These include response and collection latencies, as well as the number of correction trials and the total trials per session (e.g., if total trials are spread across a greater number of sessions for one genotype, this might bias the interpretation of reversal learning results).

Response: We thank the reviewer for bringing this to our attention. This prompted us to reevaluate our data and the statistics to validate our findings. We have re-written and re-analysed our acquisition and reversal data (section 3.8, pages 34-39) and updated Figure 11 accordingly. By examining the error types (correction and non-correction trial errors), and generating a survival curve to account for the variation in the trials over sessions between animals to reach criterion performance, we discovered that the deficit in the Irp2 ablated mice was indeed a perseverative deficit that was specific to the animals failure to

suppress their responding to the previously learned stimulus. We can also confirm that there were no differences in latencies to collect reward (Fgenotype (1,19) = 3.123, ns) or make a response (Fgenotype (1,19) = 0.418, ns) We have added the non-significant latency statistics on page 38, and updated the discussion on pages 42-43).

Minor points:

5) As body weight is used as a covariate, and was a statistically significant regressor in the motor analyses, it is important to include graphs and analyses of the raw body weights taken in parallel with the motor testing.

Response: Graphs and analysis of body weights for the rotarod and hangtime testing have been added to supplementary figure S2.

6) What were the total activity and number of entries into the open/closed sections of the elevated zero maze? These will help better interpret performance on this test.

Response: Figure 6 shows that activity and number of entries into open arms of the elecated zero maze did not differ between the two groups. Threw a some evidence that the Irp2 null mice travelled more in the closed arms. Overall, the data reveal that the mice are reduced in their anxiety. These metrics have been added to Figure 6 and commented on page 27-28.

7) Figures 4B and 8A in which significance stars are positioned over individual data points suggesting a significant interaction, however, no significant interaction effect was observed in the ANOVA. These should be removed, and the appropriate main effects indicated instead.

Response: Significance stars have been removed and main effects indicated in text. Figure 8A has been replaced.

8) Was sphericity measured and accounted for in the repeated measures ANOVA data presented in the manuscript.

Response: When data sets significantly violated the homogeneity requirement for a repeated measures design, the Geisser-Greenhouse was used to calculate a more conservative p value for each F ratio. We have dded this to the data anlaysis section (page 16).

Reviewer 2:

The authors characterized the Irp2 ablation mice in different genetic background. It is worth further clarifying the importance of IRP2 in the CNS system since IRP2 gene mutation causes a complex neurological disorder. The methods cover the various aspects concerning CNS functions. It is great. However, the manuscript can be improved. The major concerns are:

1. The manuscript is too long, and feel very redundant. Make it short and concise.

2. There is a lot of discussion in Results. Please remove it.

3. The style is oral and casual English. Make it somehow more formal.

4. For significance, $p<0.05$ is considered significant. Therefore, many sentences must be softened, removing the "strong" and other too-strong words.

Reviewer 2. We thank the reviewer for these constructive comments. We agree and have made significant changes by cutting out superfluous text, re-writing various aspects of the methods and results, the latter in line with the comments received from Reviewer #1, and corrected the English to make it professional rather than casual. We have also toned down the strength of our effects, especially those that fail to strictly meet the significance level of $p < 0.05$.

5. Mitochondrial dysfunction is supposed to be a big problem in Irp2 ablation mice and patients. This mechanism can be discussed more.

Response: We agree with the reviewer. We have alluded to this problem in the manuscript with respect to other studies that have created their own mouse models and the associated mitochondrial morphology (e.g., page 5) and have mentioned it again in the discussion (pages 44-43). Unfortunately, for this study, mitochondrial function was not systemically examined in our mouse model although this is a topic of future exploration.

The minors are:

1. In Results: mistakenly paragraphed twice.

2. Many "have/had" can be replaced by "show/showed". This might be casual-English problems.

3. The manuscript is easy to follow but has many language errors.

Response: We apologize for these oversights. These have now been corrected.

3rd Decision letter

Reference: CRNEUR-D-23-00038
Title: Ablation of Iron Regulatory Protein 2 produces a neurological disorder characterized by motor, somatosensory, and executive dysfunction in mice*
Journal: Current Research in Neurobiology

Dear Head, Section on Human Iron Metabolism Rouault,

Thank you for submitting your manuscript to Current Research in Neurobiology.

I invite you to resubmit your manuscript after addressing the remaining reviewer comments below. It is really important to take the reviewer's points in consideration as they are intended to help to improve the quality of the manuscript. As a progressive journal if you feel that you have addressed the concerns please detail this in the reply to the remaining concerns.
I look forward to receiving your revised manuscript by **Jul 18, 2024**.

When revising your manuscript, please consider all issues mentioned in the reviewers' comments carefully: please outline every change made in response to their comments and provide suitable rebuttals for any comments not addressed. Please note that your revised submission may need to be re-reviewed.

Current Research in Neurobiology values your contribution and I look forward to receiving your revised manuscript.

Kind regards,

Christopher I. Petkov
Editor in Chief
Current Research in Neurobiology

Comments from Editors and Reviewers:

Reviewer 1:

The authors make numerous changes to improve their manuscript. However, the majority of my main concerns regarding the presentation of data that have an important bearing on interpretation and conclusions remain unaddressed. These concerns are reiterated below:

1) The authors should plot and analyze the learning curve across the 6 trials of the rotarod and analyze across groups. This will help in the interpretation for why Irp2-/- mice appear to have reduced rotarod performance but increased locomotor activity in most of the maze-based tasks. If authors are concerned about the possible impact of removing one animal, they can use a mixed effects model to analyze these data across trials.

2) The authors should include age as a factor in their analysis of the grid hang data for the reasons described in previous reviews. A generalized linear mixed effects model (GLMM) can be used to help account for unequal variances between groups. Indeed, a GLMM (e.g., with logistic or probit link) appears a better option for statistical analysis than standard ANCOVA for the grid hang data given the clear non-normality and ceiling effects in the dataset. The GLMM can include age, genotype and sex as factors as well as permit body weight as covariate.

3) There appears to be some misunderstanding regarding my concern over the nociception data. Both the hot and cold plate assays depend upon paw contact with the floor surface. Mice that exhibit locomotor hyperactivity may have less time in contact with the floor with their paws. This might account for why Irp2-/- mice appear to show reduced nociceptive responses in both assays. The side angle view is not ideal, but AnyMaze might still provide some index of movement/locomotion during the test that can be analyzed as a covariate in the analysis to help rule this out. It is difficult to interpret the hot/cold plate results without this information, and/or without additional data from other tests of nociception less sensitive to locomotor activity.

4) The authors should the plot and analyze the latency, distance, errors and proportion of mice to find the escape hole for the first time on each trial on both training and probe tests to properly interpret the data. No distance data were included and the latency and proportion finding the hole data do not include the probe trial. Without these data it is difficult to interpret the main results. Indeed, the only new data indicates a significant reduction in total errors (including before and after finding the hole for

the first time) in Irp2-/- mice only during the acquisition probe trial, but NOT during the reversal period (learning or probe) suggesting that differences between mice might be due differences present or acquired during the acquisition phase rather than reversal learning phase.

5) In the touchscreen, as reversal learning is sensitive to the amount of acquisition training, in order to assert that the reversal learning effect is in fact selective, the acquisition phase trials should be included as a factor or covariate in the analysis. The reversal learning differences can only be considered selective if there is a significant genotype x phase interaction, or significant effect of genotype in the reversal phase when the acquisition phase is included as a covariate.

3rd Author Response Letter

Response to comments from Editors and Reviewers:

Reviewer 1

We thank Reviewer 1 for the additional comments. We have addressed them below, with the referees' statements in italics followed by our response. In the manuscript, all changes to the text have been highlighted in bold.

1) The authors should plot and analyze the learning curve across the 6 trials of the rotarod and analyze across groups. This will help in the interpretation for why Irp2-/- mice appear to have reduced rotarod performance but increased locomotor activity in most of the maze-based tasks. If authors are concerned about the possible impact of removing one animal, they can use a mixed effects model to analyze these data across trials.

We recognize that the relationship between the fast fall latencies in the rotarod task and the motor deficit in the Y-Maze spontaneous alternation task is not immediately apparent. In fact, the Y-Maze was the only task which the animals showed a motoric deficit, but they remained intact in their working memory performance (Fig. 7). In the Barnes maze, the deficit was due to inefficient search strategies (Fig. 8), and in the elevated and zero mazes, again, there was no evidence of increased locomotor activity (Fig. 6). Thus, motor deficits in Irp2-/- mice are not equivalent in all contexts. In the context of the additional maze data, the most parsimonious interpretation for the fast fall latencies and the increased activity in the Y-Maze is impulsivity. We have now clarified this part of the discussion (page 40).

2) The authors should include age as a factor in their analysis of the grid hang data for the reasons described in previous reviews. A generalized linear mixed effects model (GLMM) can be used to help account for unequal variances between groups. Indeed, a GLMM (e.g., with logistic or probit link) appears a better option for statistical analysis than standard ANCOVA for the grid hang data given the clear non-normality and ceiling effects in the dataset. The GLMM can include age, genotype and sex as factors as well as permit body weight as covariate.

We recognize the reviewers concern with statistical methodology especially when effect sizes are small. We performed the statistics with the resources we had and presented additional Hangtime data accounting for weight, sex and age in the supplementary materials (Fig. S2) as requested by the previous reviewer. We note that although a generalized linear mixed effects model is another option, the ANCOVA is still valid

for the data we present. In addition, as with all graphs presented in the paper, we made sure to present individual animal data to the show the variation around the mean (Fig S2).

3) There appears to be some misunderstanding regarding my concern over the nociception data. Both the hot and cold plate assays depend upon paw contact with the floor surface. Mice that exhibit locomotor hyperactivity may have less time in contact with the floor with their paws. This might account for why Irp2-/- mice appear to show reduced nociceptive responses in both assays. The side angle view is not ideal, but AnyMaze might still provide some index of movement/locomotion during the test that can be analyzed as a covariate in the analysis to help rule this out. It is difficult to interpret the hot/cold plate results without this information, and/or without additional data from other tests of nociception less sensitive to locomotor activity.

Apologies for misunderstanding the original comment. It makes sense that hyperactive mice would have less paw contact with the floor surface which might explain the somatosensory deficit observed in this test. However, if we have understood correctly, our data show the opposite effect (Fig. 3). If anything, the Irp2 ablated mice had ‘more’ contact with the floor surface since they jumped significantly less and were slow to jump (long latencies). In addition, they licked their paws less suggesting that the paws remained on the floor surface, which is consistent with their long latencies to lick. Accordingly, increased animal activity is unlikely to explain the somatosensory impairment.

4) The authors should the plot and analyze the latency, distance, errors and proportion of mice to find the escape hole for the first time on each trial on both training and probe tests to properly interpret the data. No distance data were included and the latency and proportion finding the hole data do not include the probe trial. Without these data it is difficult to interpret the main results. Indeed, the only new data indicates a significant reduction in total errors (including before and after finding the hole for the first time) in Irp2-/- mice only during the acquisition probe trial, but NOT during the reversal period (learning or probe) suggesting that differences between mice might be due differences present or acquired during the acquisition phase rather than reversal learning-phase.

We draw the reviewer’s attention to Supplementary Figure S6 which contains all these measures. We have also updated Fig. 8A to show that all mice reached the target hole in both probe trials.

5) In the touchscreen, as reversal learning is sensitive to the amount of acquisition training, in order to assert that the reversal learning effect is in fact selective, the acquisition phase trials should be included as a factor or covariate in the analysis. The reversal learning differences can only be considered selective if there is a significant genotype x phase interaction, or significant effect of genotype in the reversal phase when the acquisition phase is included as a covariate.

We agree with this overall interpretation. There is indeed a main effect of genotype during reversal when accounting for phase in the model. It remains accurate to say that there was only a within-condition effect during the reversal phases. To put it another way, the difference in the retention phase drives the overall genotype 'main effect'. The KO animals are largely clustered with WT animals in the acquisition phase but form an overall higher grouping during reversal. The prevalence of higher slopes in

KO vs. WT suggests an interaction that we lack the statistical power to resolve. We agree with the reviewer's interest in statistical rigor, but to call this a main effect without qualification would misrepresent the observation.

Accept Letter

Dear Head, Section on Human Iron Metabolism Rouault,

Thank you for submitting your manuscript to Current Research in Neurobiology.

I am pleased to inform you that your manuscript has been accepted for publication.

My comments, and any reviewer comments, are below.

Your accepted manuscript will now be transferred to our production department. We will create a proof which you will be asked to check, and you will also be asked to complete a number of online forms required for publication. If we need additional information from you during the production process, we will contact you directly.

We appreciate and value your contribution to Current Research in Neurobiology. We regularly invite authors of recently published manuscript to participate in the peer review process. If you were not already part of the journal's reviewer pool, you have now been added to it. We look forward to your continued participation in our journal, and we hope you will consider us again for future submissions.

CRNEUR aims to be a unique, community-led journal, as highlighted in the Editorial Introduction. As part of this vision, we will be regularly seeking input from the scientific community and encourage you and your co-authors to take the survey.

We would also like to invite you to take part in our CRNEUR Author Question & Answer (Q&A), which could get published alongside your article and help to promote it. We suspect you might have an interesting story of perseverance or team work that was required for the research study to complete, or a diversity of perspectives that you might share, as a way of inspiring others about neuroscience.

Kind regards,

Christopher I. Petkov
Editor in Chief
Current Research in Neurobiology

-------- End of Review Comments --------
